# Synthesis and Evaluation of ^68^Ga-Labeled (2*S*,4*S*)-4-Fluoropyrrolidine-2-Carbonitrile and (4*R*)-Thiazolidine-4-Carbonitrile Derivatives as Novel Fibroblast Activation Protein-Targeted PET Tracers for Cancer Imaging

**DOI:** 10.3390/molecules28083481

**Published:** 2023-04-14

**Authors:** Shreya Bendre, Zhengxing Zhang, Nadine Colpo, Jutta Zeisler, Antonio A. W. L. Wong, François Bénard, Kuo-Shyan Lin

**Affiliations:** 1 Department of Molecular Oncology, BC Cancer Research Institute, Vancouver, BC V5Z 1L3, Canada; sbendre@bccrc.ca (S.B.); ncolpo@bccrc.ca (N.C.); jzeisler@bccrc.ca (J.Z.); antwong@bccrc.ca (A.A.W.L.W.); fbenard@bccrc.ca (F.B.); 2 Department of Functional Imaging, BC Cancer, Vancouver, BC V5Z 4E6, Canada; 3 Department of Radiology, University of British Columbia, Vancouver, BC V5Z 1M9, Canada

**Keywords:** fibroblast activation protein α (FAP-α), cancer-associated fibroblasts (CAFs), FAP inhibitors (FAPIs), PET imaging, Gallium-68, targeted radiopharmaceuticals

## Abstract

Fibroblast activation protein α (FAP-α) is a cell-surface protein overexpressed on cancer-associated fibroblasts that constitute a substantial component of tumor stroma and drive tumorigenesis. FAP is minimally expressed by most healthy tissues, including normal fibroblasts. This makes it a promising pan-cancer diagnostic and therapeutic target. In the present study, we synthesized two novel tracers, [^68^Ga]Ga-SB03045 and [^68^Ga]Ga-SB03058, bearing a (2*S*,4*S*)-4-fluoropyrrolidine-2-carbonitrile or a (4*R*)-thiazolidine-4-carbonitrile pharmacophore, respectively. [^68^Ga]Ga-SB03045 and [^68^Ga]Ga-SB03058 were evaluated for their FAP-targeting capabilities using substrate-based in vitro binding assays, and in PET/CT imaging and ex vivo biodistribution studies in an HEK293T:hFAP tumor xenograft mouse model. The IC_50_ values of ^nat^Ga-SB03045 (1.59 ± 0.45 nM) and ^nat^Ga-SB03058 (0.68 ± 0.09 nM) were found to be lower than those of the clinically validated ^nat^Ga-FAPI-04 (4.11 ± 1.42 nM). Contrary to the results obtained in the FAP-binding assay, [^68^Ga]Ga-SB03058 demonstrated a ~1.5 fold lower tumor uptake than that of [^68^Ga]Ga-FAPI-04 (7.93 ± 1.33 vs. 11.90 ± 2.17 %ID/g), whereas [^68^Ga]Ga-SB03045 (11.8 ± 2.35 %ID/g) exhibited a tumor uptake comparable to that of [^68^Ga]Ga-FAPI-04. Thus, our data suggest that the (2*S*,4*S*)-4-fluoropyrrolidine-2-carbonitrile scaffold holds potential as a promising pharmacophore for the design of FAP-targeted radioligands for cancer diagnosis and therapy.

## 1. Introduction

Fibroblast activation protein α (FAP-α) is a type II transmembrane protein belonging to the family of non-classical serine proteases, and it cleaves Pro–Xaa peptide bonds [1]. FAP is a member of the “DPPIV-like” (DPP, dipeptidyl peptidase) class of enzymes, and possesses both dipeptidyl aminopeptidase and endopeptidase activities [1,2]. FAP is overexpressed on the cell surface of cancer-associated fibroblasts (CAFs), a type of continuously activated fibroblasts. CAFs constitute a dominant stromal component accounting for up to 80% of all stromal fibroblasts in the tumor microenvironment [2]. This overexpression is seen in the reactive stromal fibroblasts of most carcinomas, including prostate and breast cancers [3,4]. Studies have linked this overexpression to enhanced tumorigenic potential, tumor growth rates, and poor prognosis, with the association being more prominent if overexpression is found in the cancer cells rather than in the stroma [5,6]. In addition to CAFs, high levels of FAP have also been noted at tissue remodeling interfaces of cirrhotic liver [7], fibrotic lungs [8], rheumatoid arthritis synovium [9], and near sites of wound healing [10]. Interestingly, FAP is only minimally expressed in most mammalian healthy adult tissues, including normal fibroblasts [11]. FAP’s restricted tissue distribution pattern makes it a highly promising cancer diagnostic marker and therapeutic target. In this regard, several research groups have explored strategies for treating various tumors by targeting FAP with small molecule inhibitors [12,13,14,15,16], antibodies [17,18], prodrugs based on FAP’s DPP activity [19,20], CAR T-cells [21,22], and FAP vaccines [23], the latter being due to the genetic stability of CAFs over cancer cells, making it a viable cancer therapeutic target.

More recently, Haberkorn et al. synthesized and evaluated several radiolabeled small-molecule FAP inhibitors (FAPIs) for PET/CT imaging [24,25,26]. These radiolabeled FAPIs are based on a (4-quinolinoyl)-glycyl-2-cyanopyrrolidine scaffold previously reported by Jansen et al. [14]. Among these inhibitors, [^68^Ga]Ga-FAPI-04, bearing a 4,4-difluoro substitution on the (2*S*)-2-pyrrolidinecarbonitrile moiety, was able to detect several highly prevalent human cancers [27]. In a preliminary dosimetry study, in two patients [^68^Ga]Ga-FAPI-04 demonstrated a rapid tumor uptake, fast clearance via the kidneys, and very low tracer uptake in normal organs [28]. However, in the same study, considerable tracer clearance from the tumors was reported, with tumor uptake dropping from 1 to 3 h post-injection (pi) by 25%.

Jansen et al. presented a structure-activity relationship (SAR) of numerous (4-quinolinoyl)-glycyl-2-cyanopyrrolidine-based small molecules [14]. The (4*S*)-fluoropyrrolidine (IC_50_ = 3.3 nM) was reported to display comparable FAP-inhibitory potency to its 4,4-difluorinated congener (IC_50_ = 3.2 nM, found in FAPI-04). Strikingly, the (4*R*)-fluoropyrrolidine isomer (IC_50_ = 1000 nM) was found to lose its ability to inhibit FAP. This prompted us to generate our first variant, SB03045, bearing a (2*S*,4*S*)-4-fluoropyrrolidine-2-carbonitrile FAP-binding pharmacophore. We hypothesized that removal of the detrimental (4*R*)-fluoro substituent from the pyrrolidine ring of (2*S*)-4,4-difluoropyrrolidine-2-carbonitrile framework could yield a (4*S*)-mono-fluorinated version of FAPI-04 with superior FAP-inhibitory potency and, thus, demonstrate better FAP-mediated tumor uptake.

Additionally, several 4-cyanothiazolidide moiety bearing compounds were reported to be potent inhibitors of dipeptidyl peptidase-IV (DDP-IV) (K_i_ ≤ 5 nM), a serine protease that is phylogenetically related to FAP [29]. We decided to exploit this premise to generate our second variant, SB03058, bearing a (4*R*)-thiazolidine-4-carbonitrile FAP-binding pharmacophore. Retention of the N-(4-quinolinoyl)-glycyl fragment in SB03058, we reasoned, would impart FAP selectivity over DPP-IV by the virtue of the former’s endopeptidase ability.

**Figure 1:**
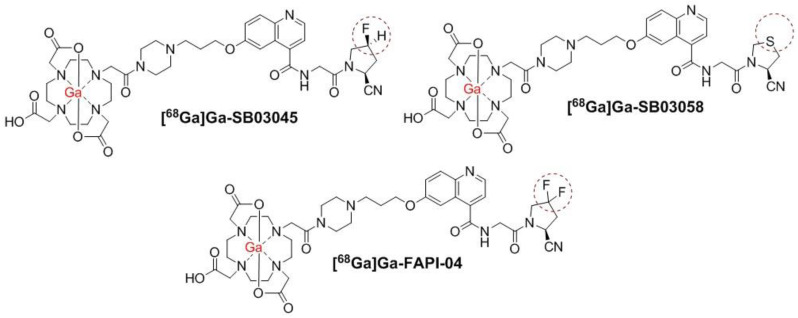
Chemical structures of ^68^Ga-labeled SB03045, SB03058 and FAPI-04. The radiolabels are in red. The differences between these three chemical structures are indicated in the brown dashed circles.

Thus, in the current work, we briefly report the design, synthesis, and evaluation of two novel FAP-targeted ligands, [^68^Ga]Ga-SB03045 and [^68^Ga]Ga-SB03058, bearing a (2*S*,4*S*)-4-fluoropyrrolidine-2-carbonitrile pharmacophore and a (4*R*)-thiazolidine-4-carbonitrile pharmacophore, respectively (Figure 1). Removal of (4*R)*-fluoro in the (2*S*)-4,4-difluoropyrrolidine-2-carbonitrile moiety of [^68^Ga]Ga-FAPI-04 yielded [^68^Ga]Ga-SB03045 with a (2*S*,4*S*)-4-fluoropyrrolidine-2-carbonitrile moiety. On the other hand, substitution of the pyrrolidine ring in [^68^Ga]Ga-FAPI-04 with a thiazolidine ring generated [^68^Ga]Ga-SB03058 with a (4*R*)-thiazolidine-4-carbonitrile scaffold. Both of the aforementioned ligands retained the (6-(3-(piperazin-1-yl)propoxy)quinoline-4-carbonyl)glycyl fragment optimized by Haberkorn et al. [24,25,26] and found in most radiolabeled FAPIs, including [^68^Ga]Ga-FAPI-04. Addition of the DOTA chelator allowed formation of stable complexes with ^68^Ga for diagnostic PET imaging. Thus, the present study is an attempt to develop radiolabeled FAPIs based on novel pharmacophores and to compare them with the clinically validated [^68^Ga]Ga-FAPI-04.

In this regard, ^nat^Ga-complexed SB03045 and SB03058 were evaluated in vitro using a substrate-based fluorescence assay to determine their FAP-inhibitory capabilities. Their ^68^Ga complexed analogs, on the other hand, were subjected to PET/CT imaging and ex vivo biodistribution studies using an HEK293T:hFAP tumor xenograft mouse model, and LogD_7.4_ partitioning studies. The results were then compared with those obtained using [^68^Ga]Ga-FAPI-04, either reported previously [30] or head-to-head in the present study.

## 2. Results

### 2.1. Synthesis of ^68^Ga and ^nat^Ga-Complexed DOTA-Conjugated FAP-Targeted Ligands

Detailed information on the synthesis, purification, and characterizations of precursors and ^nat^Ga-complexed is provided in the Supplementary Materials (Figures S1–S6).

**Scheme 1:**
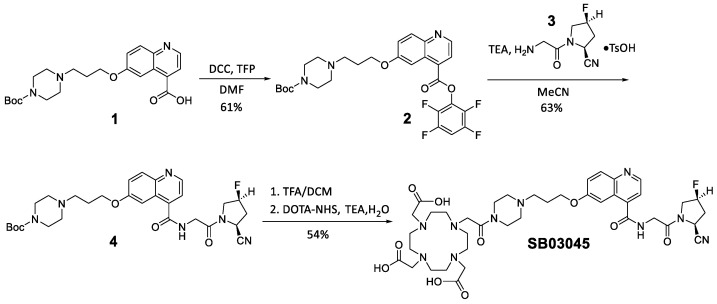
Synthesis of DOTA-conjugated precursor SB03045.

**Scheme 2:**
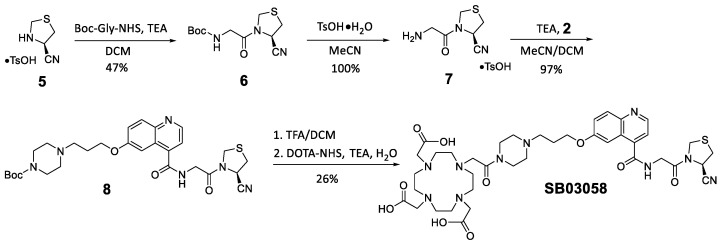
Synthesis of DOTA-conjugated precursor SB03058.

Multistep organic syntheses of DOTA-conjugated precursors SB03045 and SB03058 are presented in Scheme 1 and Scheme 2, respectively. Briefly, Compound **1** was prepared following literature procedures [24] and reacted with 2,3,5,6-tetrafluorophenol (TFP) to obtain its activated TFP-ester **2** in 61% yield [31]. Compound **2** and Compound **3** [14], prepared following literature procedures, were coupled overnight to obtain compound **4** in 63% yield. Compound **4** was first Boc-deprotected using 50% trifluoroacetic acid (TFA) in dichloromethane (DCM), then reacted with DOTA-NHS to afford the desired precursor SB03045 in 54% yield.

For the synthesis of SB03058 (Scheme 2), compound **5**, prepared following literature procedures [32], was first coupled with Boc-Gly-NHS to afford compound **6** in 47% yield. Boc deprotection of compound **6** with excess toluenesulfonic acid in acetonitrile (MeCN or ACN) yielded compound **7** quantitatively. The coupling of compound **7** with compound **2** provided compound **8** in 97% yield. Compound **8** was first Boc-deprotected using 50% TFA in DCM, then coupled with DOTA-NHS to afford precursor SB03058 in 26% yield. 

^nat^Ga-complexed standards ^nat^Ga-SB03045, ^nat^Ga-SB03058, and ^nat^Ga-FAPI-04 were obtained in 52–76% yields after incubating their respective precursors with excess ^nat^GaCl_3_ in NaOAc buffer (0.1 M, pH 4.5), followed by HPLC purification [33]. ^68^Ga labeling was performed in HEPES buffer (2 M, pH 5.0) [33], and after HPLC purification, the desired ^68^Ga-labeled analogs were obtained in 28–55% decay-corrected radiochemical yields with >98% radiochemical purity (Figures S7–S9). The molar activities were found to be ≥46 GBq/µmol for [^68^Ga]Ga-FAPI-04, ≥148 GBq/µmol for [^68^Ga]Ga-SB03045, and ≥216 GBq/µmol for [^68^Ga]Ga-SB03058.

### 2.2. In Vitro Fluorescence-Based Binding Assay

**Figure 2:**
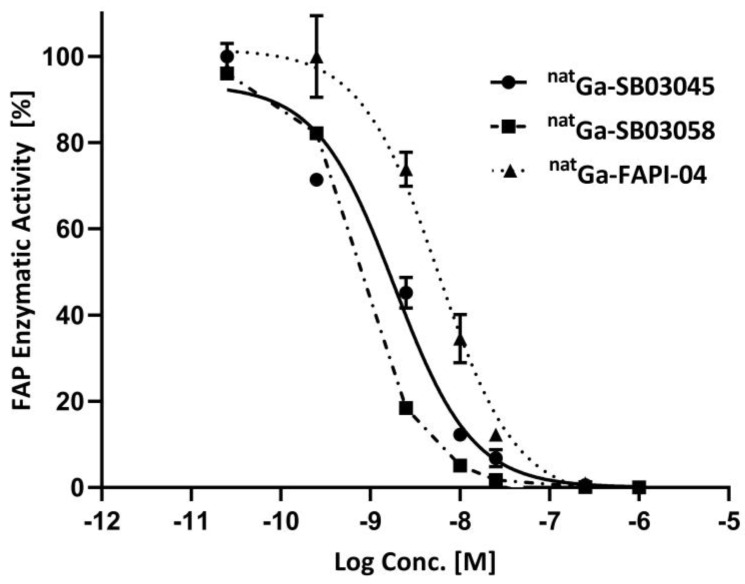
Inhibition of FAP-mediated cleavage of Suc-Gly-Pro-AMC substrate by various concentrations of ^nat^Ga-SB03045, ^nat^Ga-SB03058, and ^nat^Ga-FAPI-04.

The human FAP enzymatic activity was inhibited by ^nat^Ga-SB03045, ^nat^Ga-SB03058 and ^nat^Ga-FAPI-04 in a dose-dependent manner (Figure 2). The calculated IC_50_ values for ^nat^Ga-SB03045, ^nat^Ga-SB03058, and ^nat^Ga-FAPI-04 were 1.59 ± 0.45, 0.68 ± 0.09, and 4.11 ± 1.42 nM, respectively (n = 3).

### 2.3. Ex Vivo Biodistribution and PET/CT Imaging Studies

**Figure 3:**
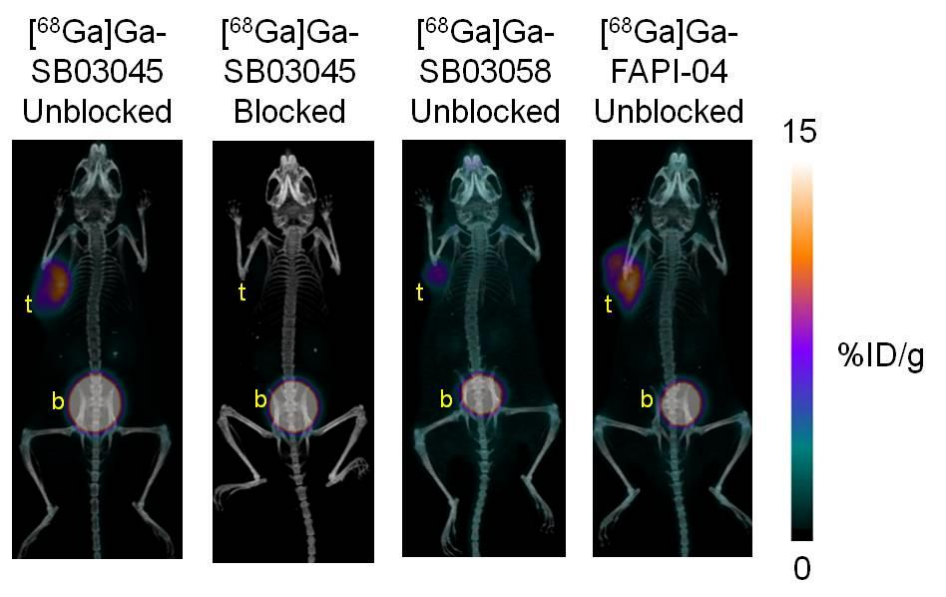
Representative maximum intensity projection PET/CT images of [^68^Ga]Ga-SB03045 (unblocked and blocked) and [^68^Ga]Ga-SB03058 at 1 h pi in HEK293T:hFAP tumor-bearing male NRG mice. The mice in the blocked group were co-injected with ^nat^Ga-FAPI-4 (0.5 mg/mouse). The image of [^68^Ga]Ga-FAPI-04 has been reported previously [30] and is displayed here for comparison. t: tumor; b: urinary bladder.

The HEK293T:hFAP tumor xenografts [30] were clearly visualized in PET/CT images acquired at 1 h pi using both [^68^Ga]Ga-SB03045 and [^68^Ga]Ga-SB03058 (Figure 3). Both tracers were excreted mainly via the renal pathway, as was apparent from the high accumulation in the urinary bladder. The tumor uptake of [^68^Ga]Ga-SB03058 was found to be lower, whereas that of [^68^Ga]Ga-SB03045 was found to be comparable to that of the previously reported [^68^Ga]Ga-FAPI-04 [30].

Although both tracers displayed fast clearance from most normal organs/tissues, the background uptake of [^68^Ga]Ga-SB03058 was slightly higher (primarily in the skeletal muscles and bones) than that of [^68^Ga]Ga-SB03045, resulting in a slightly inferior tumor/background contrast. Co-injection of [^68^Ga]Ga-SB03045 with ^nat^Ga-FAPI-04 (0.5 mg/mouse) reduced the tumor uptake to almost background level (Figure 3). 

**Figure 4:**
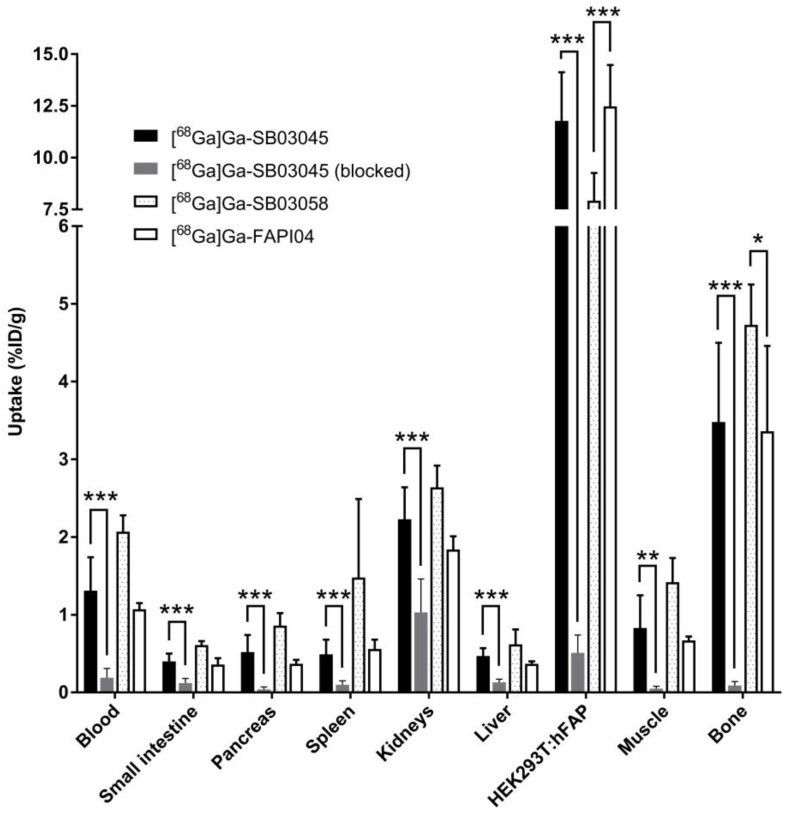
Ex vivo biodistribution of [^68^Ga]Ga-SB03045, [^68^Ga]Ga-SB03058, and [^68^Ga]Ga-FAPI-04 in HEK293T:hFAP tumor-bearing male NRG mice at 1 h pi. The mice in the blocked group were co-injected with ^nat^Ga-FAPI-4 (0.5 mg/mouse). * *p* < 0.05, ** *p* < 0.01, *** *p* < 0.001.

**Figure 5:**
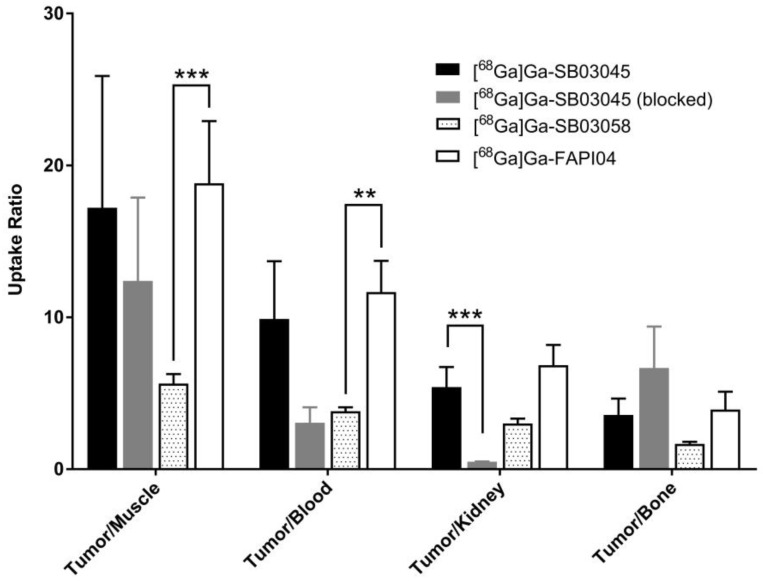
Tumor/organ uptake ratios of [^68^Ga]Ga-SB03045, [^68^Ga]Ga-SB03058, and [^68^Ga]Ga-FAPI-04 in HEK293T:hFAP tumor-bearing male NRG mice at 1 h pi. The mice in the blocked group were co-injected with ^nat^Ga-FAPI-4 (0.5 mg/mouse). ** *p* < 0.01, *** *p* < 0.001.

Biodistribution studies (Figure 4 and Table S1) conducted at 1 h pi in HEK293T:hFAP tumor-bearing mice with [^68^Ga]Ga-SB03045, [^68^Ga]Ga-SB03058 and the clinically validated [^68^Ga]Ga-FAPI-04 were found to be concordant with their PET/CT images. The tumor uptake values were 11.8 ± 2.35, 7.93 ± 1.33 and 11.9 ± 2.17 %ID/g for [^68^Ga]Ga-SB03045, [^68^Ga]Ga-SB03058 and [^68^Ga]Ga-FAPI-04, respectively. Both [^68^Ga]Ga-SB03045 and [^68^Ga]Ga-SB03058 were found to undergo renal excretion with minimal uptake in most normal organs, except bones (3.48 ± 1.02 %ID/g for [^68^Ga]Ga-SB03045 and 4.73 ± 0.52 %ID/g for [^68^Ga]Ga-SB03058), where the uptake was significantly higher than the background muscle uptake (0.83 ± 0.42 %ID/g for [^68^Ga]Ga-SB03045 and 1.42 ± 0.31 %ID/g for [^68^Ga]Ga-SB03058). The high bone uptake was also observed for [^68^Ga]Ga-FAPI-04 (bone: 3.79 ± 1.36 %ID/g; muscle: 0.76 ± 0.20 %ID/g). Compared with [^68^Ga]Ga-SB03045 and [^68^Ga]Ga-FAPI-04, [^68^Ga]Ga-SB03058 had slightly higher uptake in normal organs/tissues (Table S1). The tumor-to-muscle, tumor-to-blood, tumor-to-kidney, and tumor-to-bone uptake ratios were 17.2 ± 8.68, 9.89 ± 3.80, 5.41 ± 1.32, and 3.58 ± 1.07, respectively, for [^68^Ga]Ga-SB03045, 5.64 ± 0.62, 3.82 ± 0.25, 3.01 ± 0.32, and 1.67 ± 0.13, respectively, for [^68^Ga]Ga-SB03058, and 16.8 ± 5.73, 10.5 ± 3.25, 6.41 ± 1.52, and 3.49 ± 1.41, respectively, for [^68^Ga]Ga-FAPI-04 (Figure 5 and Table S1). In general, [^68^Ga]Ga-SB03058 had ~1.5 fold lower tumor uptake and slightly inferior tumor/organ uptake ratios compared to that of [^68^Ga]Ga-FAPI-04, whereas [^68^Ga]Ga-SB03045 performed very similar to [^68^Ga]Ga-FAPI-04.

Co-injection of [^68^Ga]Ga-SB03045 with ^nat^Ga-FAPI-04 (0.5 mg/mouse) reduced the average tumor uptake by ~96% (from 11.8 %ID/g to 0.51 %ID/g). In addition, co-injection of [^68^Ga]Ga-SB03045 with ^nat^Ga-FAPI-04 reduced the average uptake of [^68^Ga]Ga-SB03045 in normal organs/tissues, including bones (3.48 ± 1.02 %ID/g to 0.09 ± 0.05 %ID/g, Table S1).

### 2.4. Hydrophilicty/LogD_7.4_ Measurement

The LogD_7.4_ values of [^68^Ga]Ga-FAPI-04, [^68^Ga]Ga-SB03045 and [^68^Ga]Ga-SB03058 were calculated to be −1.92 ± 0.35, −2.81 ± 0.05, and −2.49 ± 0.28 respectively (n = 3). LogD_7.4_ measurements confirmed the hydrophilic properties of both [^68^Ga]Ga-SB03045 and [^68^Ga]Ga-SB03058, and both of them were found to be more hydrophilic than [^68^Ga]Ga-FAPI-04. 

## 3. Discussion

In the present study, we synthesized two novel derivatives of the clinically validated tracer [^68^Ga]Ga-FAPI-04 and evaluated them for their in vitro and in vivo FAP-targeting capabilities. Substituting the (2*S*)-4,4-difluoro-2-pyrrolidinecarbonitrile moiety in [^68^Ga]Ga-FAPI-04 with (2*S*,4*S*)-4-fluoropyrrolidine-2-carbonitrile yielded [^68^Ga]Ga-SB03045, whereas supplanting it with a (4*R*)-thiazolidine-4-carbonitrile provided [^68^Ga]Ga-SB03058. The (6-(3-(piperazin-1-yl)propoxy)quinoline-4-carbonyl)glycyl fragment, previously optimized by Haberkorn et al. [24,26] and found in FAPI-04, was retained in both SB03045 and SB03058. Finally, the addition of chelator DOTA allowed the formation of stable complexes with the PET isotope ^68^Ga. Modification was only made either to the C4 substituent in the 2-cyanopyrrolidine ring (as in SB03045) or the C4 position of 2-cyanopyrrolidine ring (as in SB03058), as rationalized below.

Structural modeling has shown that FAP contains a well-defined, hydrophobic S1 binding pocket that prefers a P1 proline with an electrophilic 2-substituent (2-cyanopyrrolidine or 2-Boro-Pro) that can form a covalent adduct with the catalytic serine nucleophile [34]. Furthermore, the S1 pocket only tolerates P2 amino acids (Gly and D-Ala) that are small enough to evade steric clashes between FAP and P3 residue or P3 residue’s carbonyl group.

Tsai et al. evaluated several different FAP inhibitors based on a substituted 4-carboxymethylpyroglutamic acid scaffold [12]. The C2-position substituent on the lactam ring was varied and the resulting small molecules were evaluated for FAP-inhibitory properties. A 4,4-difluoro substituent at the (2*S*)-2-cyanopyrrolidine ring (IC_50_ = 22 nM) resulted in a 4-fold increase in FAP-inhibitory activity compared to that of the unsubstituted analog (IC_50_ = 79 nM). Interestingly, the removal of the (4*R*)-fluoro substituent at the (2*S*)-2-cyanopyrrolidine ring yielded an FAP inhibitor (IC_50_ = 20 nM) with an inhibitory potency that was comparable to that of the 4,4-difluoro version. Recently, a structure-activity relationship and FAP-inhibitory potencies of numerous (4-quinolinoyl)-glycyl-2-cyanopyrrolidine-based small molecules have been reported [13,14]. The effect of substituting the C4 position of the 2-cyanopyrrolidine moiety was thoroughly showcased. In the study, (4*S*)-fluoropyrrolidine (IC_50_ = 3.3 nM) was reported to exhibit FAP affinity that was comparable to that of the 4,4-difluorinated substitution (IC_50_ = 3.2 nM, found in FAPI-04) and 3-fold higher FAP affinity than that of the unsubstituted analog (IC_50_ = 10.3 nM). Strikingly, the (4*R*)-fluoropyrrolidine isomer (IC_50_ = 1000 nM) was found to lose its FAP-inhibitory capabilities. Pivotal studies by Raines et al. [35] showed that whereas the 4*R* isomer possesses an unfavorable exo-puckered ring conformation, the 4*S* isomer adopts a hyperconjugatively stabilized endo-puckered ring conformation that contributes to its higher potency. This prompted us to generate our first variant, SB03045, bearing a (2*S*,4*S*)-4-fluoropyrrolidine-2-carbonitrile FAP-binding pharmacophore. We rationalized that by removing unfavorable (4*R*)-fluoro substituent at the (2*S*)-4,4-difluoropyrrolidine-2-carbonitrile moiety, the resulting (4*S*)-mono-fluorinated version of FAPI-04 may have higher FAP-inhibitory potency and exhibit better FAP-mediated tumor uptake.

A series of pseudodipeptides based on a 3-aminoacyl-4-cyanothiazolidide scaffold (K_i_ ≤ 5 nM) were reported to be very potent inhibitors of DDP-IV [29]. In particular, 3-isoleucyl-4-cyanothiazolidine (K_i_ = 0.41 nM) was demonstrated to have >5-fold higher binding affinity than its (2*S*)-2-cyanopyrrolidine counterpart (K_i_ = 2.2 nM). DPP-IV is a serine protease that hails from a class of prolyl peptidases [1,36,37]. However, unlike FAP, it is strictly a dipeptidyl exopeptidase and is only able to cleave dipeptides containing proline at the penultimate position from the *N*-terminus of peptides or proteins [1]. Accordingly, we substituted the pyrrolidine ring in FAPI-04 with a thiazolidine ring to yield our second derivative, SB03058, containing a (4*R*)-thiazolidine-4-carbonitrile binding moiety that was investigated as another novel FAP-targeted pharmacophore. We rationalized that the addition of *N*-(4-quinolinoyl)-glycyl fragment to the (4*R*)-thiazolidine-4-carbonitrile pharmacophore would impart selectivity toward FAP over its phylogenetically related dipeptidyl exopeptidase counterpart DPP-IV, which is unable to recognize *N*-blocked/acylated peptides.

The average IC_50_ value of ^nat^Ga-SB03045 (1.59 ± 0.45 nM) was marginally lower, whereas that of ^nat^Ga-SB03058 (0.68 ± 0.09 nM) was found to be less by an order of magnitude than ^nat^Ga-FAPI-04 (4.11 ± 1.42 nM) in the FAP-binding assays. Thus, the FAP-binding potency of the mono-fluorinated analog was found to be slightly better, whereas that of the thiazolidine version was observed to be >5 fold higher than the di-fluorinated analog. This confirmed our hypothesis that replacing the (2*S*)-4,4-difluoropyrrolidine-2-carbonitrile moiety in FAPI-04 with (2*S*,4*S*)-4-fluoropyrrolidine-2-carbonitrile or (4*R*)-thiazolidine-4-carbonitrile could lead to new derivatives with improved FAP-binding affinity. 

In LogD_7.4_ studies performed to determine lipophilicity, [^68^Ga]Ga-SB03045 and [^68^Ga]Ga-SB03058 were found to possess comparable hydrophilic properties (−2.81 ± 0.05 vs. −2.49 ± 0.28, respectively) and to be more hydrophilic than [^68^Ga]Ga-FAPI-04 (−1.92 ± 0.35). This was consistent with previous reports that difluorination augments hydrophobicity [14].

PET/CT images acquired at 1 h pi showed clear visualization of both [^68^Ga]Ga-SB03045 and [^68^Ga]Ga-SB03058, which were excreted mainly via the renal pathway (Figure 3). The data from ex vivo biodistribution studies performed at 1 h pi (Table S1) were concordant with the PET/CT imaging data, and both [^68^Ga]Ga-SB03045 and [^68^Ga]Ga-SB03058 revealed good FAP-targeting capabilities. Interestingly, contrary to the better in vitro FAP-inhibitory performance of ^nat^Ga-SB03058 than ^nat^Ga-FAPI-04, its radioactive equivalent [^68^Ga]Ga-SB03058 (7.93 ± 1.33 %ID/g) demonstrated a ~1.5 fold lower tumor uptake than that of [^68^Ga]Ga-FAPI-04 (11.9 ± 2.17 %ID/g). The tumor uptake of [^68^Ga]Ga-SB03045 (11.8 ± 2.35 %ID/g) was comparable to that of [^68^Ga]Ga-FAPI-04, which was consistent with the results obtained from the FAP-binding assays, where the IC_50_ values of [^68^Ga]Ga-SB03045 and [^68^Ga]Ga-FAPI-04 were found to be of the same order of magnitude. 

Although both tracers displayed fast clearance from most normal organs/tissues, the tumor/background contrast of the thiazolidine derivative [^68^Ga]Ga-SB03058 was slightly inferior to those of the mono-fluorinated [^68^Ga]Ga-SB03045 and di-fluorinated [^68^Ga]Ga-FAPI-04. In addition to a lower tumor uptake, this can be attributed to the higher background uptake of [^68^Ga]Ga-SB03058, particularly in blood and skeletal muscles (2.07 ± 0.21 and 1.42 ± 0.31 %ID/g, respectively), compared to [^68^Ga]Ga-SB03045 (1.31 ± 0.43 and 0.83 ± 0.42 %ID/g, respectively) and [^68^Ga]Ga-FAPI-04 (1.20 ± 0.30 and 0.76 ± 0.20 %ID/g, respectively). The excellent tumor-to-background contrast seen on the PET/CT image of [^68^Ga]Ga-SB03045 (Figure 3) and comparable to that previously obtained using [^68^Ga]Ga-FAPI-04 [30] was further corroborated from the correspondingly comparable tumor/organ uptake ratios obtained in biodistribution studies (Figure 5 and Table S1). [^68^Ga]Ga-SB03058, on the other hand, demonstrated a slightly inferior tumor/background than those of the mono- and di-fluorinated analogs. Thus, while (2*S*,4*S*)-4-fluoropyrrolidine-2-carbonitrile containing tracer [^68^Ga]Ga-SB03045 performed in a manner similar to that of its di-fluorinated analog, [^68^Ga]Ga-FAPI-04, it significantly outperformed the (4*R*)-thiazolidine-4-carbonitrile moiety bearing variant, [^68^Ga]Ga-SB03058.

The biodistribution data of mice co-injected with ^nat^Ga-FAPI-04 (0.5 mg/mouse) revealed a ~96% reduction in the tumor uptake and further substantiated the in vivo FAP specificity of our lead candidate [^68^Ga]Ga-SB03045. Interestingly, blocking studies also revealed the bone uptake of [^68^Ga]Ga-SB03045 to be FAP-specific, as co-injection with excess ^nat^Ga-FAPI-04 resulted in a ~97% reduction in bone uptake.

Since [^68^Ga]Ga-SB03045 exhibited low blood retention at 1 h pi (1.31 ± 0.43 %ID/g), we did not perform imaging and biodistribution studies at later time points to see if tumor uptake can be further improved over time. Instead, derivatives of [^68^Ga]Ga-SB03045 containing an albumin binder to maximize tumor uptake by virtue of extended blood residence could be exploited in the future, as similar approaches have been reported by others to improve the tumor uptake of [^68^Ga]Ga-FAPI-04 [38,39].

There are varying and contradictory reports on the association between the uptake intensity of radiolabeled FAPIs and FAP expression [25,40,41,42,43]. In general, the extent to which the preclinical mouse model is representative of what may be seen in patients and how FAP expression in normal tissues, such as multipotent bone marrow stromal cells, the cervix, and the uterus in humans, would influence the diagnostic performance and radiotherapeutic application of FAP inhibitors still remains ambiguous. More clinical studies may be required in order to ascertain these associations.

The present study design may have certain limitations. The current work is more of a preliminary study in which we were simply trying to compare the potential of two of our novel FAP-targeted pharmacophores for PET/CT imaging against FAPI-04. The data on tracer kinetics is limited, as PET/CT imaging or biodistribution studies were not carried out at multiple time points and no dynamic imaging was performed. Elaborate studies to obtain data on tracer pharmacokinetics and dosimetry to estimate absorbed radiation doses to normal organs/tissues are required in the future if our lead candidate, SB03045, is to be taken for clinical translation. Moreover, FAPI-46 was recently identified as an improvement on FAPI-04 with therapeutic capabilities, due to a longer tumor-retention time than that of FAPI-04 [25]. It would be interesting to incorporate the highly promising FAPI-46 linker into our lead candidate and evaluate the FAP-targeting capabilities of this newly generated agent against its parent compound FAPI-46.

## 4. Materials and Methods

### 4.1. Synthesis of ^nat^Ga-Complexed DOTA-Conjugated FAP-Targeted Ligands

Detailed information on the synthesis, purification, and characterizations of precursors (SB03045 and SB03058) and intermediates and their ^nat^Ga-complexed analogs (^nat^Ga-SB03045 and ^nat^Ga-SB03058) is provided in the Supplementary Materials.

### 4.2. Cell Culture

HEK293T cells were obtained from the American Type Culture Collection (Manassas, VA, USA). The IMPACT Rodent Pathogen Test (IDEXX BioAnalytics, Columbia, MO, USA) verified that the cells were pathogen-free. Detailed information on the generation of HEK293T:hFAP cells has been previously reported by our group [30]. HEK293T:hFAP cells were cultured in DMEM GlutaMAX™ medium supplemented with 10% FBS, penicillin (100 U/mL), and streptomycin (100 µg/mL) at 37 °C in a Panasonic Healthcare (Tokyo, Japan) MCO-19AIC humidified incubator containing 5% CO_2_. Cells were grown until 80–90% confluence and washed with sterile PBS (pH 7.4) and collected.

### 4.3. In Vitro Fluorescence Based Binding Assay

The half-maximal inhibitory concentration (IC_50_) values of the FAP-targeted ligands were measured using an in vitro enzymatic assay. Briefly, recombinant human FAP (Biolegend, San Diego, CA, USA; 0.2 µg/mL, 50 µL) was added into a Costar clear bottom 96-well plate. PBS and varied concentrations (25 pM to 1 µM) of ^nat^Ga-complexed ligands were added to each well (in duplicate) containing the recombinant human FAP. After being incubated for 30 min at 37 °C, Suc-Gly-Pro-AMC (Bachem, Bubendorf, Switzerland; 20 mM, 50 µL) was added to each well. The velocities of AMC release were measured kinetically at λ_ex_ = 380 nm, λ_em_ = 460 nm at 60 min at 37 °C using a FlexStation 3 Multi-Mode Microplate Reader. 

### 4.4. General Procedure for Synthesis of ^68^Ga-Complexed Radiotracers 

Following our previously published procedures [33], purified [^68^Ga]GaCl_3_ (171 to 260 MBq) in 0.55 mL water was added to a solution of 10 nmol precursor in 0.65 mL HEPES buffer (2M, pH 5.0). The reaction mixture was incubated in a Danby (Guelph, Canada) microwave oven model DMW7700WDB for 1 min at power level 2. After cooling down for 1 min at ambient temperature, the mixture was then purified using HPLC. The eluate fractions containing ^68^Ga-labeled radiotracer were collected, diluted with PBS (50 mL), and passed through a C18 Sep-Pak cartridge. ^68^Ga-labeled radiotracer trapped on the cartridge was eluted off with ethanol (containing 100 ppm ascorbic acid) and formulated with PBS (containing 100 ppm ascorbic acid) and run on HPLC for quality control before animal studies were performed. The HPLC conditions for preparation of a [^68^Ga]Ga-SB03045 were a C18 semi-prep column eluted with 10% acetonitrile (containing 0.1% TFA) and 90% deionized water (containing 0.1% TFA) at a flow rate of 4.5 mL/min, and the retention time of [^68^Ga]Ga-SB03045 was 17.6 min. The HPLC conditions for quality control of [^68^Ga]Ga-SB03045 were a C18 analytical column eluted with 14% acetonitrile (containing 0.1% TFA) and 86% deionized water (containing 0.1% TFA) at a flow rate of 2.0 mL/min, and the retention time of [^68^Ga]Ga-SB03045 was 5.6 min. The HPLC conditions for preparation of [^68^Ga]Ga-SB03058 were a C18 semi-prep column eluted with 11% acetonitrile (containing 0.1% TFA) and 89% deionized water (containing 0.1% TFA) at a flow rate of 4.5 mL/min, and the retention time of [^68^Ga]Ga-SB03058 was 16.1 min. The HPLC conditions for quality control of [^68^Ga]Ga-SB03058 were a C18 analytical column eluted with 13% acetonitrile (containing 0.1% TFA) and 87% deionized water (containing 0.1% TFA) at a flow rate of 2.0 mL/min, and the retention time of [^68^Ga]Ga-SB03058 was 7.2 min. The HPLC conditions for preparation of [^68^Ga]Ga-FAPI-04 were a C18 semi-prep column eluted with 11% acetonitrile (containing 0.1% TFA) and 89% deionized water (containing 0.1% TFA) at a flow rate of 4.5 mL/min, and the retention time of [^68^Ga]Ga-FAPI-04 was 20.1 min. The HPLC conditions for quality control of [^68^Ga]Ga-FAPI-04 were a C18 analytical column eluted with 13% acetonitrile (containing 0.1% TFA) and 87% deionized water (containing 0.1% TFA) at a flow rate of 2.0 mL/min, and the retention time of [^68^Ga]Ga-FAPI-04 was 8.0 min.

### 4.5. Ex Vivo Biodistribution and PET/CT Imaging Studies

Imaging and biodistribution studies were performed using immunodeficient male NRG (NOD.Cg-Rag1tm1Mom Il2rgtm1Wjl/SzJ) mice. All experiments were conducted according to the guidelines established by the Canadian Council on Animal Care and approved by Animal Ethics Committee of the University of British Columbia. The mice were subcutaneously inoculated with 7.5 × 10^6^ HEK293T:hFAP cells in the left dorsal flank. When the tumors grew to 6–8 mm in diameter, the mice were used for PET/CT imaging and biodistribution studies.

PET/CT imaging experiments were carried out using a Siemens Inveon micro PET/CT scanner (Knoxville, TN, USA). Tumor-bearing mice were injected with ~4 to 6 MBq (0.02–0.13 nmol) of ^68^Ga-labeled tracer through a lateral caudal tail vein under 2.5% isoflurane in oxygen anesthesia, followed by recovery and free roaming in their cage during the uptake period. A 10 min localization CT scan was acquired using 3 overlapping positions to cover each entire mouse. A CT scan was used for attenuation and scatter correction, and anatomical localization. A list mode acquisition was then performed for 15 min at 1 h pi with each mouse under isoflurane sedation. The images were reconstructed using 3D OSEM/MAP iterative methods.

For biodistribution studies, the mice were injected with ~1–2 MBq (0.01–0.04 nmol) of the ^68^Ga-labeled tracer, using the exact procedures described above. At 1 h post injection, the mice were euthanized by CO_2_ inhalation. Blood was withdrawn by cardiac puncture and organs/tissues of interest were collected, weighed, and counted using a Perkin Elmer (Waltham, MA, USA) Wizard2 2480 automatic gamma counter. The percent injected dose per gram of tissues (%ID/g) for different tracers was calculated.

For blocking studies, HEK293T:hFAP tumor-bearing mice were co-injected with [^68^Ga]Ga-SB03045 and excess nonradioactive Ga-FAPI-04 (0.5 mg/mouse) as a competitor. Imaging and biodistribution were performed at 1 h pi, similar to the process for the unblocked mice.

### 4.6. Hydrophilicty/LogD_7.4_ Measurement

Briefly, ^68^Ga-labeled tracers (1.11–1.48 MBq) were aliquoted into vials containing 3 mL of n-octanol and 3 mL of 0.1 M phosphate buffer (pH 7.4) in triplicate. Each vial was then vortexed (1 min) and centrifuged (5000 RPM, 10 min). The n-octanol and aqueous layers were sampled (1 mL) and counted using an automated gamma counter and the LogD_7.4_ value was calculated using the following equation:

$${LogD}_{7.4}={log}_{10}\left[\left(\mathrm{counts}\;\mathrm{in}\;\text{n}-\mathrm{octanol}\;\mathrm{phase}\right)/\left(\mathrm{counts}\;\mathrm{in}\;\mathrm{buffer}\;\mathrm{phase}\right)\right]$$

### 4.7. Statistical Analysis

Data were reported as mean ± standard deviation (SD) and analyzed with GraphPad Prism, version 7.02, and Microsoft (Redmond, WA, USA) Excel. One-way ANOVA and multiple *t*-tests were performed for all tumor and organ/tissue uptake values in the biodistribution studies of [^68^Ga]Ga-SB03045, [^68^Ga]Ga-SB03058, and [^68^Ga]Ga-FAPI-04 in the HEK293T:hFAP tumor model. Statistical significance was defined at *p* < 0.05 using the Holm–Sidak method.

## 5. Conclusions

Replacing the (2*S*)-4,4-difluoro-2-pyrrolidinecarbonitrile moiety in [^68^Ga]Ga-FAPI-04 with (2*S*,4*S*)-4-fluoropyrrolidine-2-carbonitrile or (4*R*)-thiazolidine-4-carbonitrile yielded [^68^Ga]Ga-SB03045 and [^68^Ga]Ga-SB03058, respectively, which were successfully synthesized and evaluated in a preclinical tumor model. Both derivatives retained good binding affinity to FAP. While the substitution with (4*R*)-thiazolidine-4-carbonitrile reduced tumor uptake and tumor-to-background contrast ratios, the replacement with (2*S*,4*S*)-4-fluoropyrrolidine-2-carbonitrile moiety retained good tumor uptake and tumor-to-background contrast ratios. In this work, we found (2*S*,4*S*)-4-fluoropyrrolidine-2-carbonitrile scaffold to be a promising pharmacophore for the design of FAP-targeted radioligands for cancer diagnosis. Future optimization by the addition of albumin binding moieties, in order to increase its blood residence time and maximize tumor uptake, is warranted, especially for the design of radiotherapeutic agents to treat FAP-expressing tumors.

## Data Availability

The data generated from this study are available in the text and in the Supplementary Materials.

## Supplementary Materials

The following supporting information can be downloaded at: https://www.mdpi.com/article/10.3390/molecules28083481/s1. Detailed synthetic procedures and results for the preparation of FAP-targeted ligands and their ^nat^Ga-complexed analogs; Table S1: Biodistribution and tumor/organ uptake ratios of [^68^Ga]Ga-SB03045, [^68^Ga]Ga-SB03058 and [^68^Ga]Ga-FAPI-04 in HEK239T:hFAP tumor-bearing mice; Figure S1: A representative MS spectrum of FAPI-04; Figure S2: A representative MS spectrum of SB03045; Figure S3: A representative MS spectrum of SB03058; Figure S4: A representative MS spectrum of ^nat^Ga-FAPI-04; Figure S5: A representative MS spectrum of ^nat^Ga-SB03045; Figure S6: A representative MS spectrum of ^nat^Ga-SB03058; Figure S7: Radio-HPLC analysis of [^68^Ga]Ga-FAPI-04; Figure S8: Radio-HPLC analysis of [^68^Ga]Ga-SB03045; Figure S9: Radio-HPLC analysis of [^68^Ga]Ga-SB03058.
