# Supplementary material for: Synthesis and Evaluation of 68Ga-Labeled (2S,4S)-4-Fluoropyrrolidine-2-Carbonitrile and (4R)-Thiazolidine-4-Carbonitrile Derivatives as Novel Fibroblast Activation Protein-Targeted PET Tracers for Cancer Imaging
Source: Molecules. 2023 Apr 14;28(8):3481. doi: 10.3390/molecules28083481 (PMC10145249; doi:10.3390/molecules28083481)
Supplement: Supplementary file 1 [file molecules-28-03481-s001.zip › molecules-2321516-supplementary.pdf]

## SUPPLEMENTARY MATERIALS

### Detailed synthetic procedures and results for the preparation of FAP-targeted ligands and their natGa-complexed analogs

### General methods

6-(3-(4-(*Tert*-butoxycarbonyl)piperazin-1-yl)propoxy)quinoline-4-carboxylic acid (**1**), (2*S*,4*S*)-4-fluoro-1-glycylpyrrolidine-2-carbonitrile (**3**), FAPI-04 and (*R*)-thiazolidine-4-carbonitrile (**5**) were prepared following literature procedures [1-3]. All other chemicals were procured from commercial sources and used without further purification. Purification and quality control of radiolabeling precursors, nonradioactive Ga-complexed standards and $^{68}$Ga-labeled tracers were performed on Agilent (Santa Clara, CA) HPLC systems equipped with a model 1200 quaternary pump, a model 1200 UV absorbance detector (set at 220 nm), and a Bioscan (Washington, DC) NaI scintillation detector. The operation of Agilent HPLC systems was controlled using the Agilent ChemStation software. HPLC columns used were a semi-preparative column (Luna C18, 5 μm particle size, 100 Å pore size, 250 × 10 mm) and an analytical column (Luna C18, 5 μm particle size, 100 Å pore size, 250 × 4.6 mm) from Phenomenex (Torrance, CA). The collected HPLC eluates containing the desired products were lyophilized using a Labconco (Kansas City, MO) FreeZone 4.5 Plus freeze drier. Mass analyses were performed using a Waters (Milford, MA) Acquity QDa mass spectrometer with the equipped 2489 UV/Vis detector and e2695 Separations module. C18 Sep-Pak cartridges (1 cm$^3$, 50 mg) were obtained from Waters (Milford, MA). $^{68}$Ga was eluted from an ITM Medical Isotopes GmbH (Munich, Germany) generator and purified according to the previously published procedures using a DGA resin column from Eichrom Technologies LLC (Lisle, IL) [4]. Radioactivity of radiolabeled ligands was measured using a Capintec (Ramsey, NJ) CRC-25R/W dose calibrator. The radioactivity of mouse tissues collected from biodistribution studies was counted using a PerkinElmer (Waltham, MA) Wizard2 2480 automatic gamma counter.

### Synthesis of DOTA-conjugated FAP-targeting ligands

<u>Synthesis of 2,3,5,6-tetrafluorophenyl 6-(3-(4-(*tert*-butoxycarbonyl)piperazin-1-yl)propoxy)quinoline-4-carboxylate (**2**)</u>

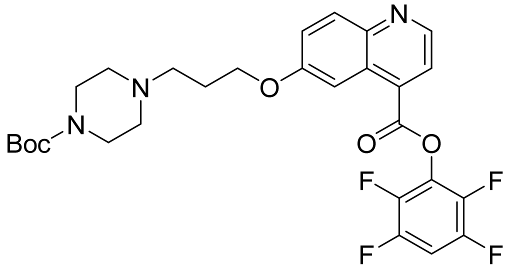


Dicyclohexylcarbodiimide (1.62 g, 7.9 mmol) was added to a solution of compound **1** (3.12 g, 7.5 mmol) and 2,3,5,6-tetrafluorophenol (1.49 g, 9.0 mmol) in 60 mL of *N*,*N*-dimethylformamide. The resulting solution was stirred at room temperature overnight and filtered. The filtrate was evaporated and purified by flash column chromatography using 100% ethyl acetate as the eluent to obtain 2.58 g (61% yield) of compound **2** as a light yellow solid. ESI-MS: calculated $[M+H]^+$ for $C_{28}H_{29}F_4N_3O_5$ 564.2 found 564.1. $^1$H NMR

(300 MHz, $CDCl_3$) δ 9.00 (d, *J* = 4.5 Hz, 1H), 8.23 (d, *J* = 4.4 Hz, 1H), 8.20 (d, *J* = 2.7 Hz, 1H), 8.14 (d, *J* = 9.3 Hz, 1H), 7.46 (dd, *J* = 9.2, 2.7 Hz, 1H), 7.13 (tt, *J* = 9.9, 7.1 Hz, 1H), 4.24 (t, *J* = 5.8 Hz, 2H), 3.73 (s, 4H), 3.64–3.03 (br, 2H), 2.36 (s, 4H), 1.48 (s, 9H).

Synthesis of (2*S*,4*S*)-4-fluoro-1-glycylpyrrolidine-2-carbonitrile *p*-toluenesulfonate salt (**3**)

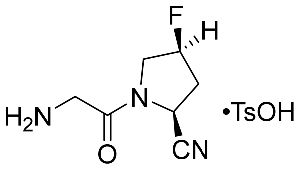


Compound **3** was synthesized following literature procedures [1]. Briefly, commercially available (2*S*,4*S*)-*tert*-butyl 2-cyano-4-fluoropyrrolidine-1-carboxylate (2.48 g, 11.6 mmol) was Boc-deprotected with *p*-toluenesulfonic acid monohydrate (3.33 g, 17.5 mmol) in $CH_3CN$ (30 mL) at room temperature overnight. The reaction was evaporated, and the residue was stirred with ether (250 mL) for 1 h, filtered and dried under reduced pressure to give 3.43 g of crude (2*S*,4*R*)-4-fluoropyrrolidine-2-carbonitrile *p*-toluenesulfonate salt as a grey solid which was used without further purification.

A mixture of crude (2*S*,4*R*)-4-fluoropyrrolidine-2-carbonitrile *p*-toluenesulfonate salt (591 mg), 8.6 mmol), Boc-glycine *N*-hydroxysuccinimide ester (Boc-Gly-OSu, 327 mg, 1.2 mmol) and triethylamine (304 mg, 3.0 mmol) in $CH_2Cl_2$ (10 mL) was stirred at room temperature overnight. After evaporation, the residue was dissolved in ethyl acetate (100 mL) and the resulting solution was washed with 1N HCl (50 mL × 2), dried over anhydrous $MgSO_4$, filtered and evaporation under reduced pressured to yield 303 mg of crude Boc-protected (2*S*,4*S*)-4-fluoro-1-glycylpyrrolidine-2-carbonitrile as a light yellow solid which was used without further purification.

Crude Boc-protected (2*S*,4*S*)-4-fluoro-1-glycylpyrrolidine-2-carbonitrile (303 mg) was Boc-deprotected with *p*-toluenesulfonic acid monohydrate (342 mg, 1.8 mmol) in $CH_3CN$ (10 mL) at room temperature overnight. The reaction was evaporated to yield the crude (2*S*,4*S*)-4-fluoro-1-glycylpyrrolidine-2-carbonitrile *p*-toluenesulfonate salt **3** which was used without further purification.

Synthesis of *tert*-butyl 4-(3-((4-((2-((2*S*,4*S*)-2-cyano-4-fluoropyrrolidin-1-yl)-2-oxoethyl)carbamoyl)quinolin-6-yl)oxy)propyl)piperazine-1-carboxylate (**4**)

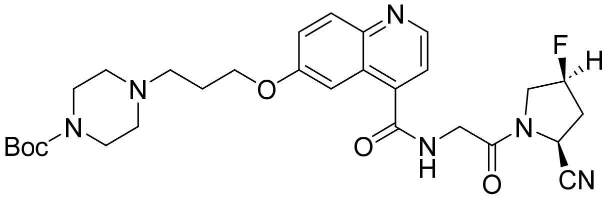


A mixture of compound **3** (294 mg, 1.1 mmol) and triethylamine (506 mg, 5.0 mmol, 697 uL) were dissolved in $CH_3CN$ (10 mL). Compound **2** (456 mg, 0.81 mmol) was dissolved in $CH_3CN$ (10 mL) and added to it. The reaction was stirred overnight at room temperature. After evaporation, the residue was purified by flash column chromatography using 1:5 methanol/ethyl acetate to obtain 288 mg (63% yield) of compound **4** as a light purple solid. ESI-MS: calculated $[M+H]^+$ for $C_{29}H_{37}FN_6O_5$ 569.3 found 569.5. $^1H$ NMR (300 MHz, $CDCl_3$) δ 8.81 (dd, *J* = 4.4, 2.1 Hz, 1H), 8.04 (d, *J* = 9.2 Hz, 1H), 7.71 (d, *J* = 2.7 Hz, 1H), 7.50 (d, *J* = 4.4 Hz, 1H), 7.41 (dd, *J* = 9.3, 2.7 Hz, 1H), 7.17 (s, 1H), 5.51 (dt, *J* = 50.4, 3.5 Hz, 1H), 4.99 (d, *J* = 9.1 Hz, 1H), 4.49 – 3.95

(m, 5H), 3.47 (t, *J* = 5.1 Hz, 4H), 2.78 (q, *J* = 14.8 Hz, 1H), 2.61 (q, *J* = 7.5 Hz, 2H), 2.49 (d, *J* = 7.0 Hz, 4H), 2.18 (s, 1H), 2.15 – 2.02 (m, 1H), 1.47 (s, 9H), 1.27 (d, *J* = 1.7 Hz, 1H).

Synthesis of DOTA-conjugated precursor SB03045

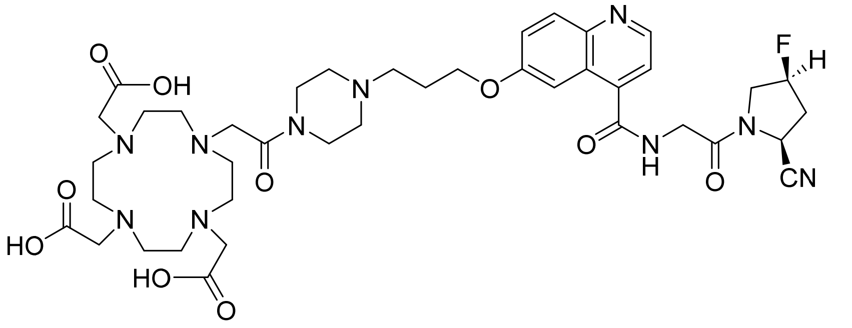

Boc-removal of compound **4** (20 mg, 35 μmol) was completed with 2 mL 1:1 (v/v) TFA/$CH_2Cl_2$ for 1 h at room temperature. The solvent was removed *in vacuo* and resuspended in 3 mL 2:1 (v/v) $H_2O/CH_3CN$. The resulting solution was neutralized by dropwise addition of triethylamine. DOTA-NHS (40 mg, 53 μmol) was added and the reaction was stirred overnight at room temperature. The crude mixture was purified with HPLC (C18 semi-prep column, 4.5 mL/min, 10% $CH_3CN$ (0.1%TFA), retention time: 9.0 min). The elution fractions containing the desired product were collected and lyophilized to give a white powder. Yield: 54%. ESI-MS: calculated $[M+H]^+$ for SB03045 $C_{40}H_{55}FN_{10}O_{10}$ 855.4; found 855.3.

Synthesis of (*R*)-thiazolidine-4-carbonitrile *p*-toluenesulfonate salt (**5**)

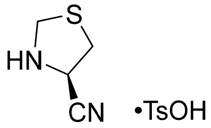

Following literature procedures [3], deprotection of Boc-protected (*R*)-thiazolidine-4-carbonitrile (2.47 g, 11.5 mmol) proceeded with *p*-toluenesulfonic acid monohydrate (3.33 g, 17.5 mmol) in $CH_3CN$ (30 mL) at room temperature overnight. The reaction was evaporated, and the residue was stirred with ether (250 mL) for 1 h, filtered and dried under reduced pressure to give 3.81 g of crude compound **5** (contaminated with TsOH) as a white solid which was used without further purification.

Synthesis of *tert*-butyl (*R*)-(2-(4-cyanothiazolidin-3-yl)-2-oxoethyl)carbamate (**6**)

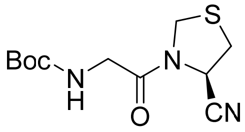

A mixture of compound **5** (2.48 g, 8.6 mmol) and triethylamine (2.02 g, 20.0 mmol, 2.79 mL) in $CH_2Cl_2$ (30 mL) was stirred at room temperature for a few minutes before adding Boc-glycine *N*-hydroxysuccinimide ester (Boc-Gly-OSu) (1.36 g, 5.0 mmol). The reaction was stirred at room temperature for 2 days and evaporated. The residue was purified by flash column chromatography using 1:2 ethyl acetate/hexanes to give 634 mg (47% yield) of compound **6** as a white solid. ESI-MS: calculated $[M+H]^+$ for $C_{11}H_{17}N_3O_3S$ 272.1;

found 272.2. $^{1}$H NMR (300 MHz, $CDCl_3$) δ 5.34 – 5.25 (m, 2H), 4.56 (s, 2H), 4.13 – 3.88 (m, 2H), 3.29 (d, *J* = 4.5 Hz, 2H), 1.45 (s, 9H).

Synthesis of (*R*)-3-glycylthiazolidine-4-carbonitrile *p*-toluenesulfonate salt (**7**)

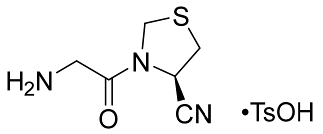


Boc-removal of compound **6** (610 mg, 2.2 mmol) proceeded with *p*-toluenesulfonic acid monohydrate (647 mg, 3.4 mmol) in $CH_3CN$ (15 mL) at room temperature overnight. The reaction was evaporated to give residue that was stirred with ether (250 ml) for 1 h, filtered and dried under reduced pressure to give 1.12 g (100%) of compound **7** as a tosylate salt without further purification.

Synthesis of *tert*-butyl (*R*)-4-(3-((4-((2-(4-cyanothiazolidin-3-yl)-2-oxoethyl)carbamoyl)quinolin-6-yl)oxy)propyl)piperazine-1-carboxylate (**8**)

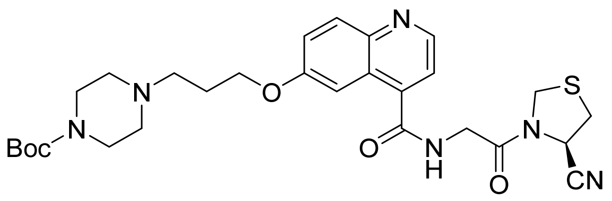


A mixture of compound **7** (1.12 g, 2.2 mmol) and triethylamine (405 mg, 4.0 mmol, 558 uL) was dissolved in $CH_3CN$ (5 mL) and $CH_2Cl_2$ (25 mL). Compound **2** (1.05 g, 1.86 mmol) was added to the solution. The resulting mixture was stirred overnight at room temperature. After evaporation, the residue was purified by flash column chromatography using 1:5 methanol/ethyl acetate to obtain 1.03 g (97% yield) of compound **8** as a light yellow solid. ESI-MS: calculated $[M+H]^+$ for $C_{28}H_{36}N_6O_5S$ 569.3; found 569.5. $^{1}$H NMR (300 MHz, $CDCl_3$) δ 8.82 (d, *J* = 4.4 Hz, 1H), 8.05 (d, *J* = 9.2 Hz, 1H), 7.68 (d, *J* = 2.6 Hz, 1H), 7.51 (d, *J* = 4.4 Hz, 1H), 7.42 (dd, *J* = 9.3, 2.7 Hz, 1H), 7.04 (s, 1H), 5.30 (t, *J* = 4.6 Hz, 1H), 4.69 (s, 2H), 4.45 (qd, *J* = 17.6, 4.5 Hz, 2H), 4.18 (q, *J* = 7.5 Hz, 2H), 3.47 (t, *J* = 5.1 Hz, 5H), 3.37 (d, *J* = 4.7 Hz, 2H), 2.60 (t, *J* = 7.3 Hz, 2H), 2.46 (t, *J* = 5.0 Hz, 4H), 2.07 (q, *J* = 6.5 Hz, 2H), 1.47 (s, 9H).

Synthesis of DOTA-conjugated precursor SB03058

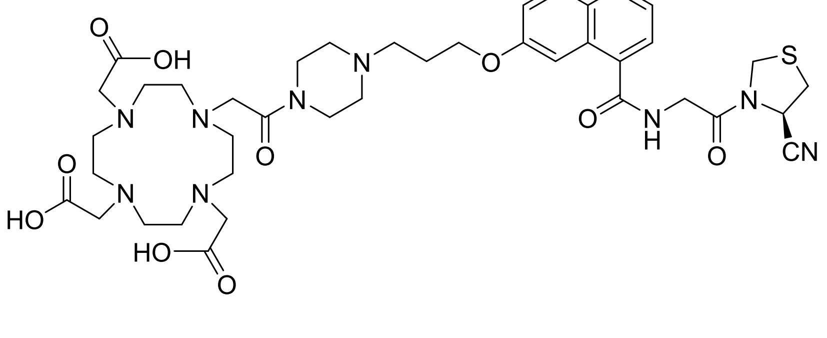

Boc-removal was completed with compound **8** (20 mg, 35 μmol) in 2 mL of 1:1 (v/v) TFA/$CH_2Cl_2$ for 1 h at room temperature. The reaction mixture was evaporated and the residue was dissolved in 3 mL 2:1 (v/v) $H_2O$/$CH_3CN$ and the resulting solution was neutralized by dropwise addition of triethylamine. DOTA-NHS (40 mg, 53 μmol) was added and the reaction mixture was stirred overnight at room temperature. The crude mixture was purified with HPLC (C18 semi-prep column, 4.5 mL/min, 11% $CH_3CN$ (0.1%TFA), retention time: 8.9 min). The elution fractions containing the desired product were collected and lyophilized to give a white powder. Yield: 26%. ESI-MS: calculated $[M+H]^+$ for SB03058 $C_{39}H_{54}N_{10}O_{10}S$ 855.4; found 855.4.

**General procedure for synthesis of $^{nat}$Ga-complexed compounds**

Following our previously published procedures [4], 2 mg of precursor was dissolved in 0.2 mL NaOAc buffer (0.1 M, pH 4.5) and $^{nat}GaCl_3$ (5 eq., 42 μL, 0.27 M) was added. The reaction mixture was incubated at 90 °C for 30 min and then purified using HPLC. The elution fractions containing the desired product were collected and lyophilized to give a white powder.

Synthesis of $^{nat}$Ga-FAPI-04

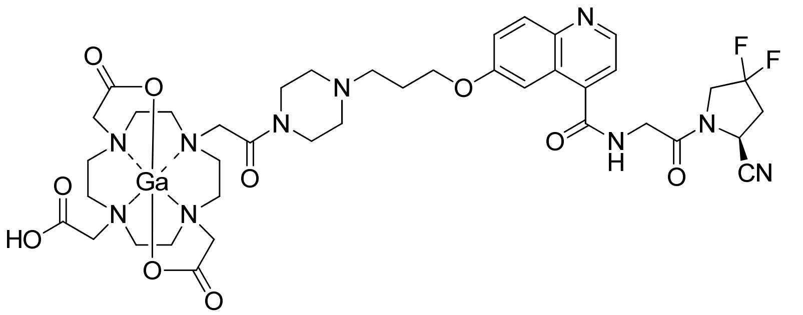

HPLC conditions used: C18 semi-prep column, 4.5 mL/min, 11% $CH_3CN$ (0.1%TFA), retention time: 12.1 min. Yield: 52%. ESI-MS: calculated $[M+2H]^{2+}$ for $^{nat}$Ga-FAPI-04 $C_{40}H_{51}F_2GaN_{10}O_{10}$ 470.7; found 470.1

Synthesis of $^{nat}$Ga-SB03045

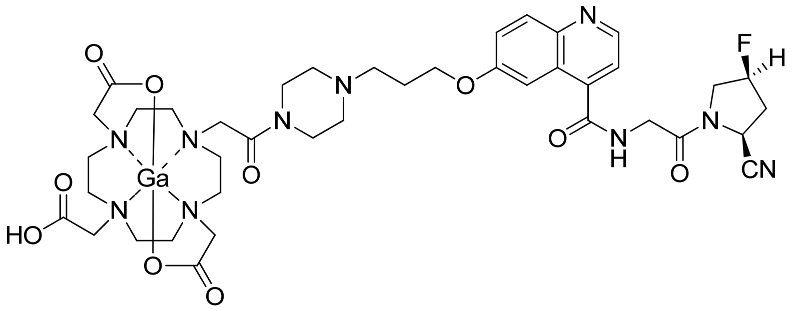

HPLC conditions used: C18 semi-prep column, 4.5 mL/min, 11% $CH_3CN$ (0.1%TFA), retention time: 11.0 min. Yield: 56%. ESI-MS: calculated $[M+2H]^{2+}$ for $^{nat}$Ga-SB03045 $C_{40}H_{52}FGaN_{10}O_{10}$ 461.7; found 461.4

Synthesis of $^{nat}$Ga-SB03058

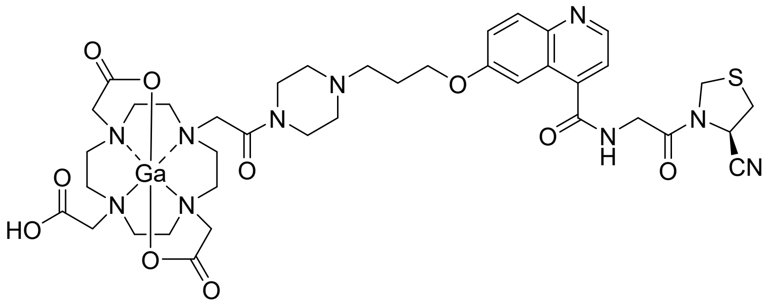


HPLC conditions used: C18 semi-prep column, 4.5 mL/min, 11% $CH_3CN$ (0.1%TFA), retention time: 9.4 min. Yield: 76%. ESI-MS: calculated $[M+2H]^{2+}$ for $^{nat}$Ga-SB03058 $C_{39}H_{51}GaN_{10}O_{10}S$ 461.6; found 461.2

**Table S1:** Biodistribution and tumor/organ uptake ratios of [$^{68}$Ga]Ga-SB03045, [$^{68}$Ga]Ga-SB03058 and [$^{68}$Ga]Ga-FAPI-04 in HEK239T:hFAP tumor-bearing mice. For blocking, [$^{68}$Ga]Ga-SB03045 was co-injected with $^{nat}$Ga-FAPI-04 (0.5 mg per mouse).

| **Tissue** | **[$^{68}$Ga]Ga-SB03045** | **[$^{68}$Ga]Ga-SB03058** | **[$^{68}$Ga]Ga-FAPI-04** | **[$^{68}$Ga]Ga-SB03045** |
|---|---|---|---|---|
| (%ID/g) | (1 h, unblocked) N=4 | (1 h, unblocked) N=4 | (1 h, unblocked) N=5 | (1 h, blocked) N=3 |
| Blood | 1.31 ± 0.43 | 2.07 ± 0.21 | 1.20 ± 0.30 | 0.19 ± 0.12 |
| Fat | 0.14 ± 0.08 | 0.40 ± 0.23 | 0.14 ± 0.06 | 0.04 ± 0.02 |
| Testes | 0.26 ± 0.08 | 0.48 ± 0.10 | 0.28 ± 0.04 | 0.06 ± 0.03 |
| Small Intestines | 0.40 ± 0.10 | 0.61 ± 0.05 | 0.37 ± 0.07 | 0.12 ± 0.06 |
| Stomach | 0.31 ± 0.04 | 0.26 ± 0.07 | 0.16 ± 0.19 | 0.07 ± 0.06 |
| Pancreas | 0.52 ± 0.22 | 0.86 ± 0.16 | 0.44 ± 0.16 | 0.04 ± 0.03 |
| Spleen | 0.49 ± 0.19 | 1.48 ± 1.01 | 0.60 ± 0.14 | 0.10 ± 0.05 |
| Adrenal Glands | 0.49 ± 0.47 | 2.50 ± 1.58 | 1.31 ± 0.60 | 0.14 ± 0.08 |
| Kidneys | 2.23 ± 0.41 | 2.64 ± 0.28 | 1.88 ± 0.18 | 1.03 ± 0.43 |
| Liver | 0.47 ± 0.10 | 0.62 ± 0.19 | 0.40 ± 0.07 | 0.13 ± 0.04 |
| Heart | 0.41 ± 0.15 | 0.59 ± 0.09 | 0.37 ± 0.08 | 0.05 ± 0.03 |
| Lungs | 0.69 ± 0.20 | 1.25 ± 0.11 | 0.80 ± 0.16 | 0.15 ± 0.09 |
| Tumor | 11.8 ± 2.35 | 7.93 ± 1.33 | 11.9 ± 2.17 | 0.51 ± 0.23 |
| Muscle | 0.83 ± 0.42 | 1.42 ± 0.31 | 0.76 ± 0.20 | 0.05 ± 0.03 |
| Bone | 3.48 ± 1.02 | 4.73 ± 0.52 | 3.79 ± 1.36 | 0.09 ± 0.05 |
| Brain | 0.05 ± 0.01 | 0.11 ± 0.08 | 0.05 ± 0.01 | 0.01 ± 0.01 |
| | | | | |
| Tumor/Muscle | 17.2 ± 8.68 | 5.64 ± 0.62 | 16.8 ± 5.73 | 12.4 ± 5.48 |
| Tumor/Blood | 9.89 ± 3.80 | 3.82 ± 0.25 | 10.5 ± 3.25 | 3.06 ± 1.01 |
| Tumor/Kidney | 5.41 ± 1.32 | 3.01 ± 0.32 | 6.41 ± 1.52 | 0.50 ± 0.01 |
| Tumor/Bone | 3.58 ± 1.07 | 1.67 ± 0.13 | 3.49 ± 1.41 | 6.67 ± 2.72 |

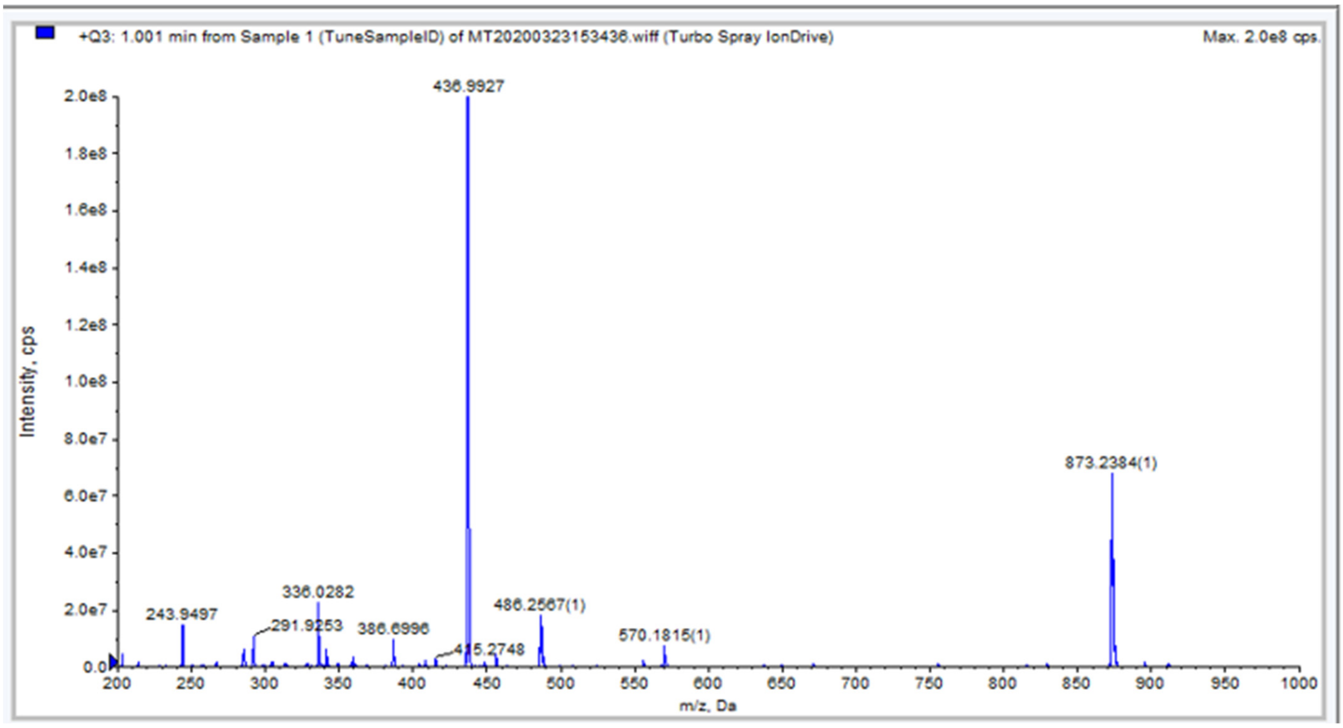


**Figure S1:** A representative MS spectrum of FAPI-04. The calculated m/z values for $[M+H]^+$ and $[M+2H]^{2+}$ are 873.4 and 437.2, respectively; observed 873.2 and 437.0, respectively.

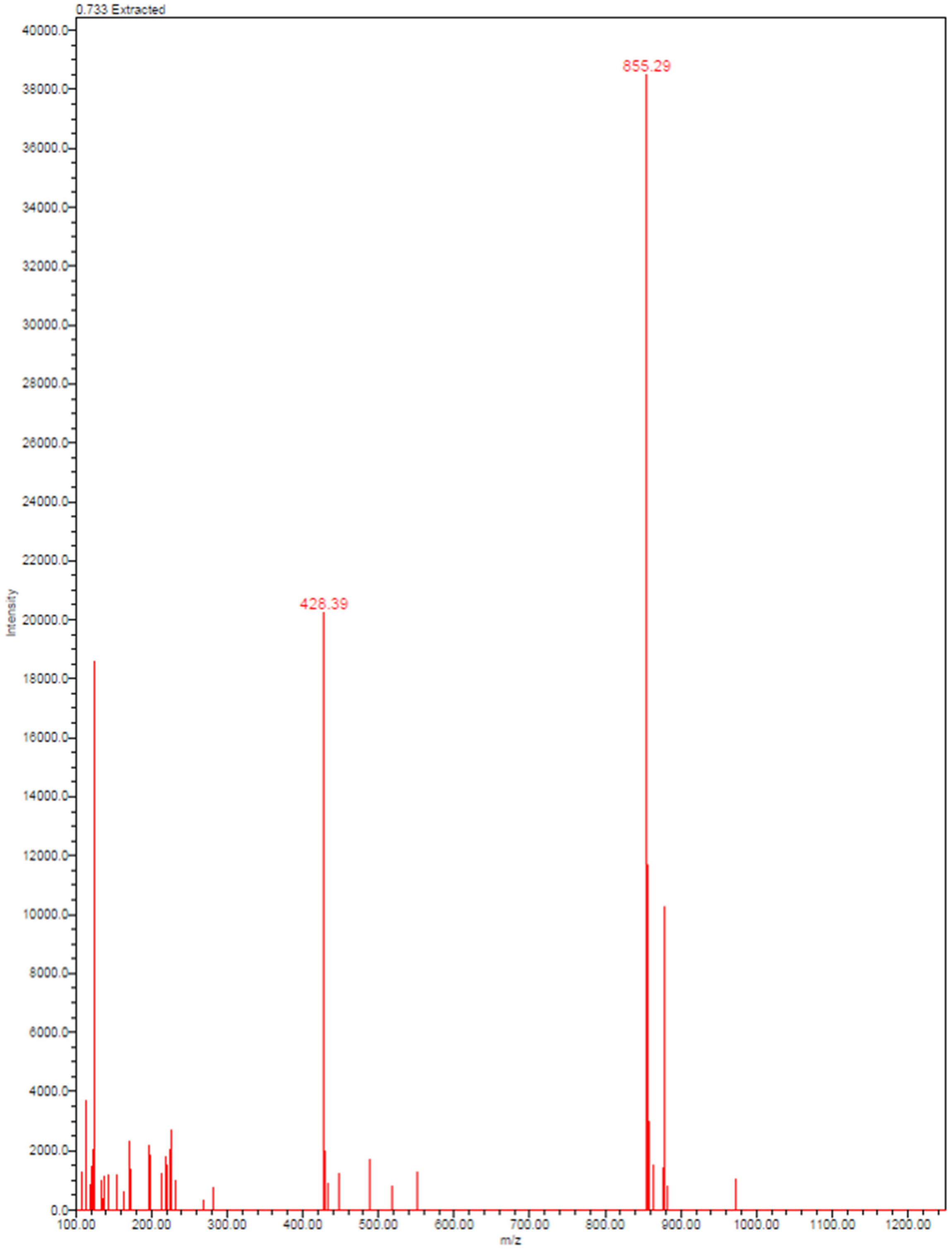


**Figure S2:** A representative MS spectrum of SB03045. The calculated m/z values for $[M+H]^+$ and $[M+2H]^{2+}$ are 855.4 and 428.2, respectively; observed 855.3 and 428.4, respectively.

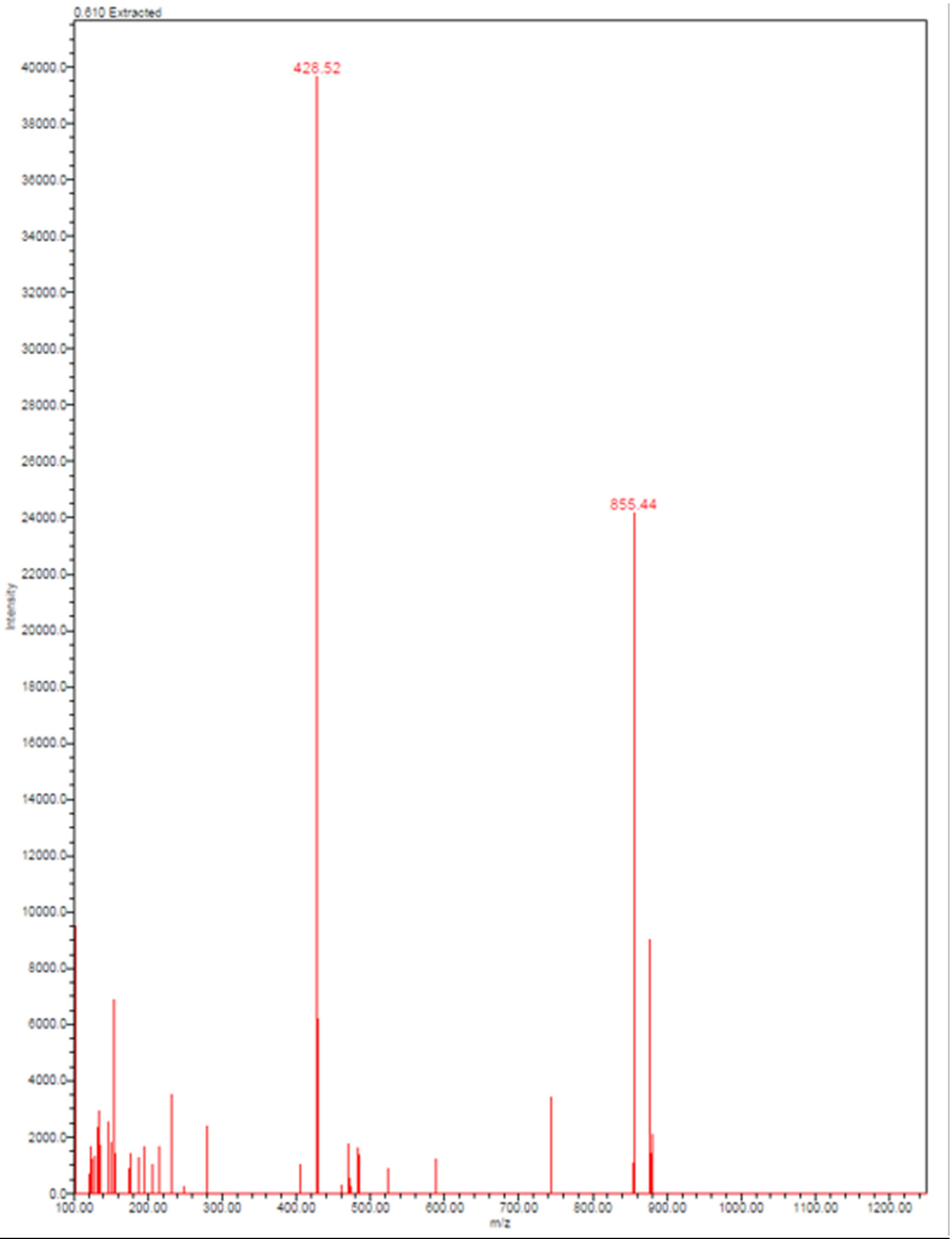


**Figure S3:** A representative MS spectrum of SB03058. The calculated m/z values for $[M+H]^+$ and $[M+2H]^{2+}$ are 855.4 and 428.2, respectively; observed 855.4 and 428.5, respectively.

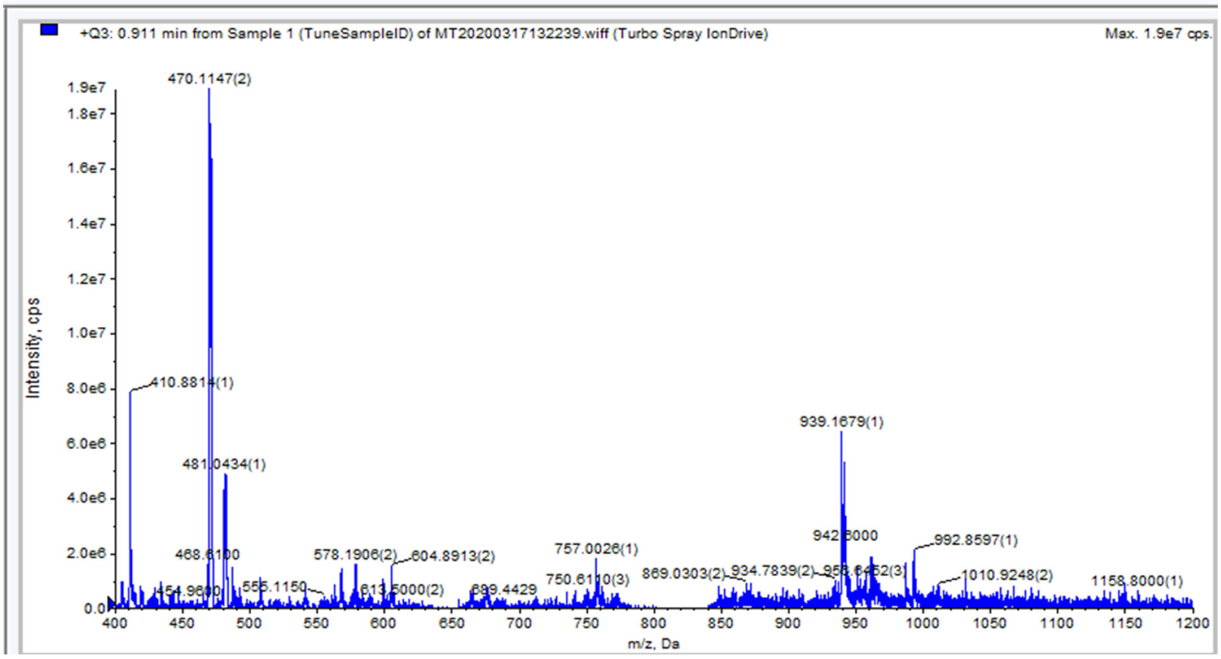


**Figure S4:** A representative MS spectrum of $^{nat}$Ga-FAPI-04. The calculated m/z values for $[M+H]^+$ and $[M+2H]^{2+}$ are 940.3 and 470.7, respectively; observed 939.2 and 470.1, respectively.

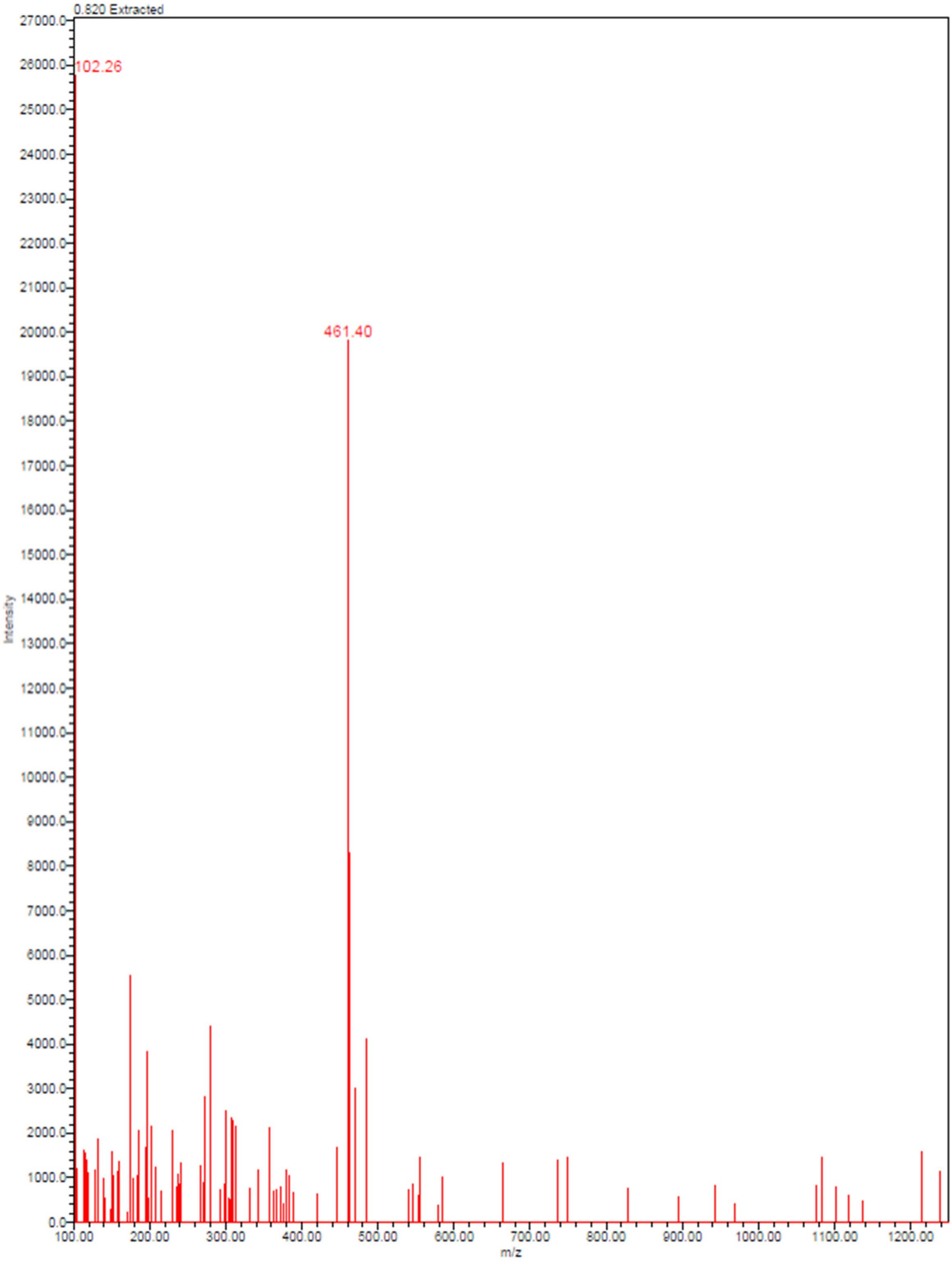


**Figure S5:** A representative MS spectrum of $^{nat}$Ga-SB03045. The calculated m/z value for $[M+2H]^{2+}$ is 461.7; observed 461.4.

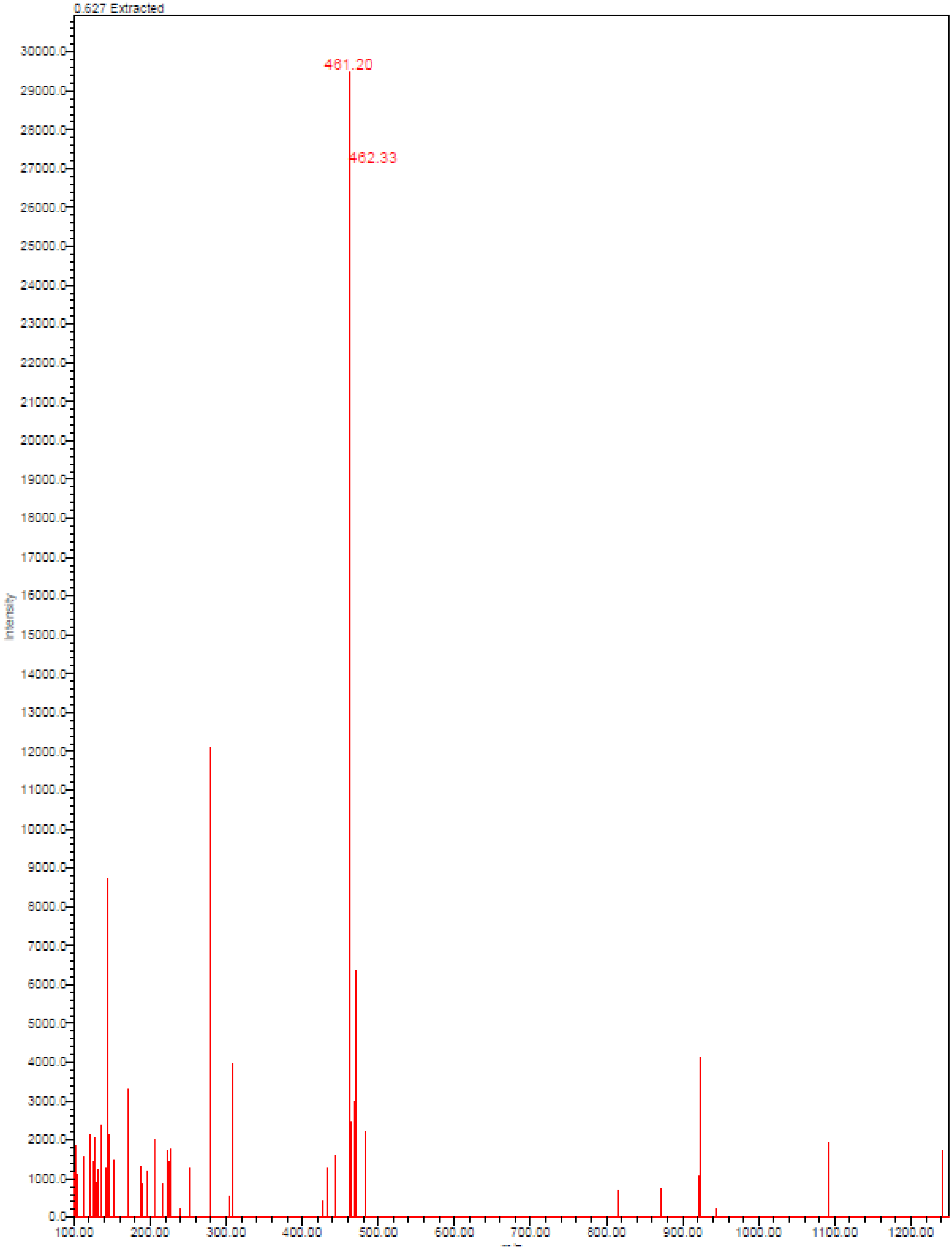


**Figure S6:** A representative MS spectrum of $^{nat}$Ga-SB03058. The calculated m/z value for $[M+2H]^{2+}$ is 461.6; observed 461.2.

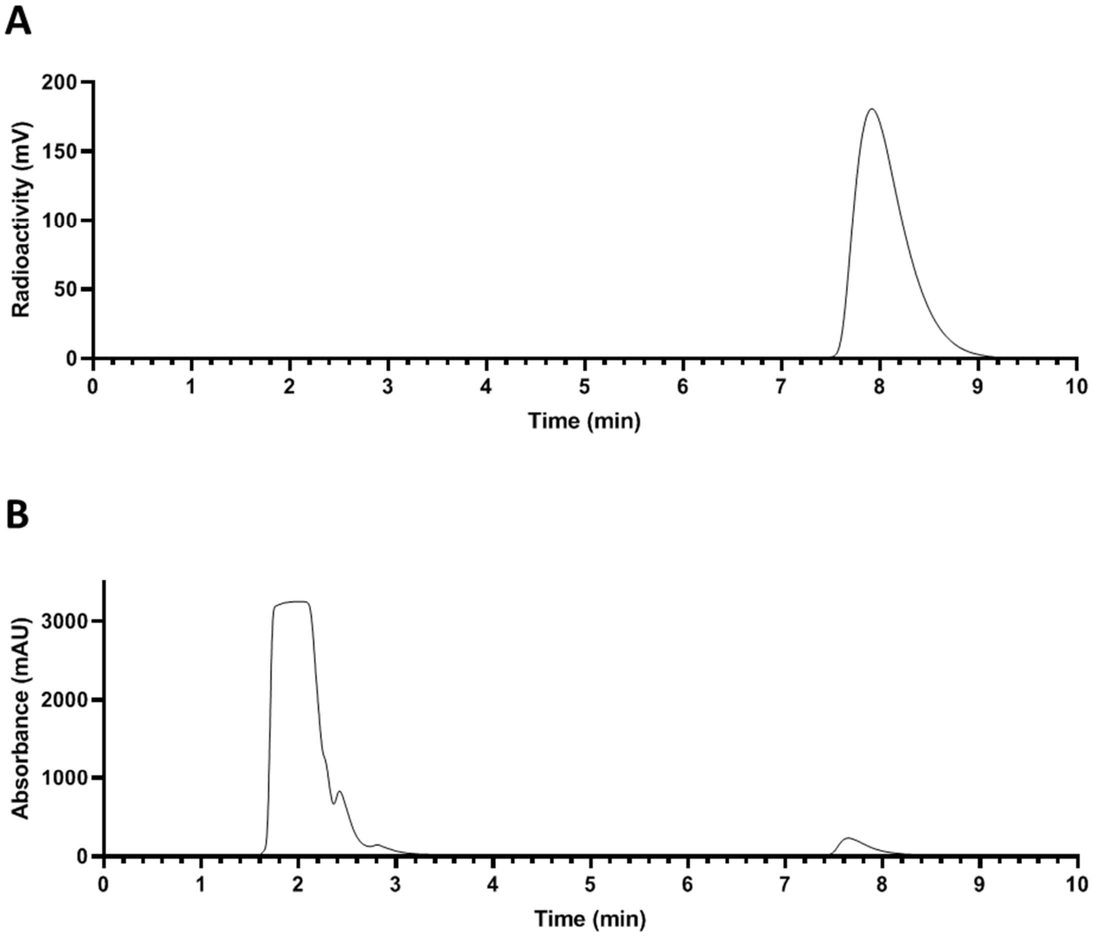


**Figure S7:** Radio-HPLC analysis of [$^{68}$Ga]Ga-FAPI-04: (A) QC radio-chromatogram (B) UV- chromatogram upon co-injection of $^{nat}$Ga-FAPI-04.

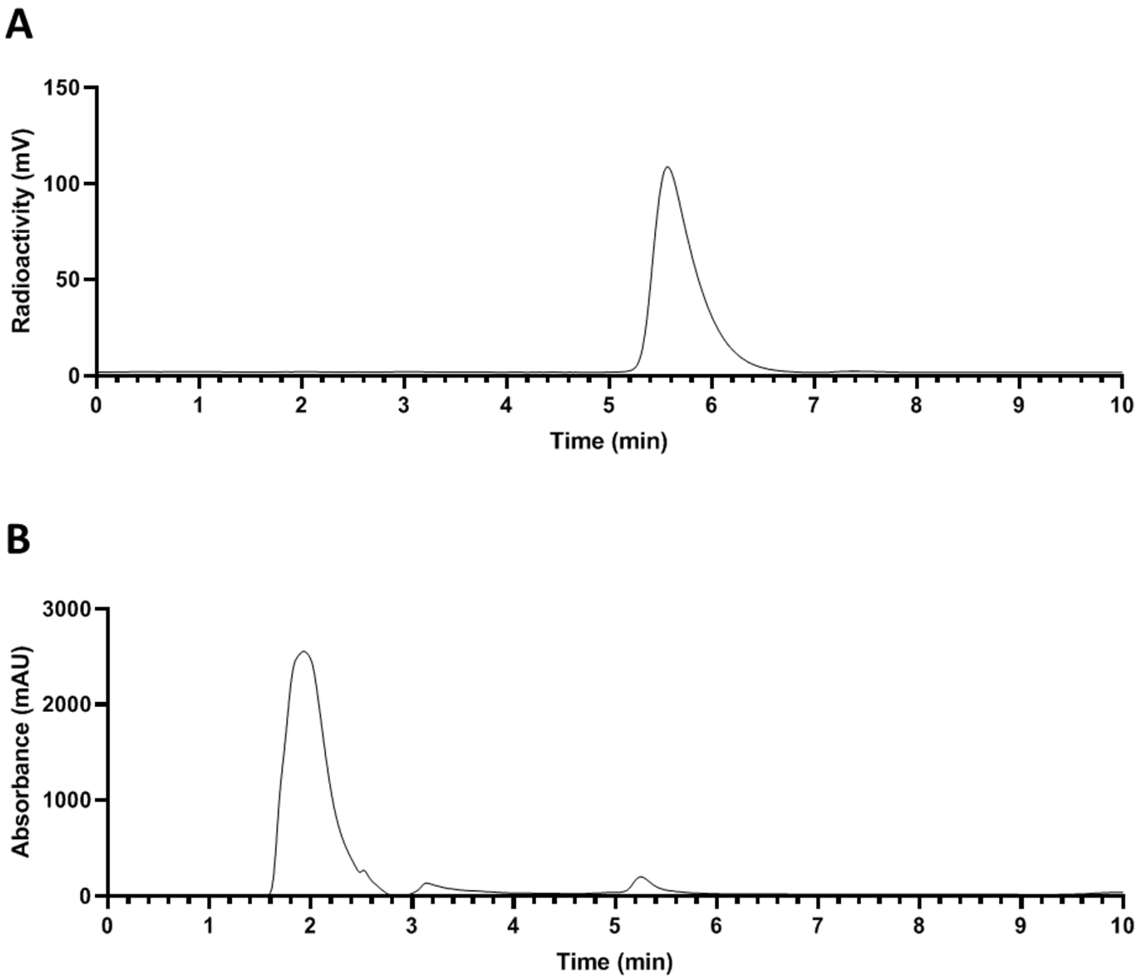


**Figure S8:** Radio-HPLC analysis of [$^{68}$Ga]Ga-SB03045: (A) QC radio-chromatogram (B) UV- chromatogram upon co-injection of $^{nat}$Ga-SB03045.

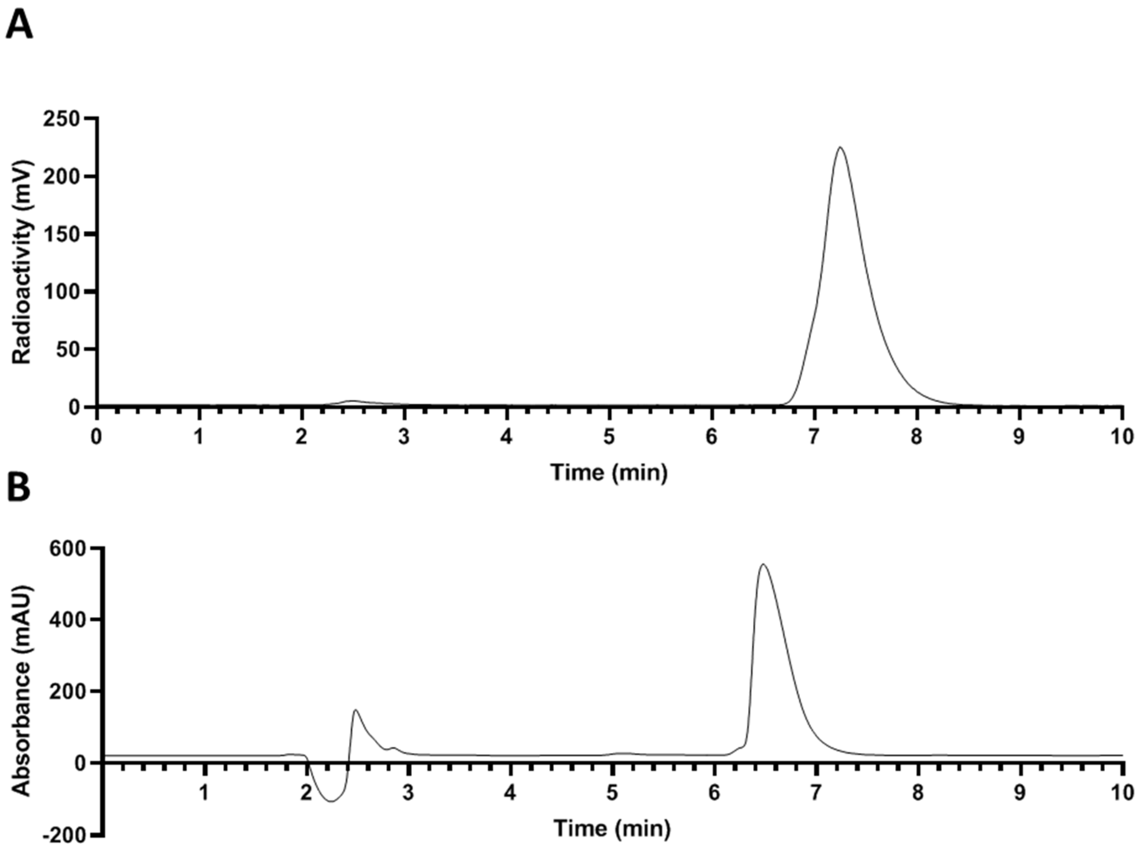


**Figure S9:** Radio-HPLC analysis of [$^{68}$Ga]Ga-SB03058: (A) QC radio-chromatogram (B) UV-chromatogram upon co-injection of $^{nat}$Ga-SB03058.

**References:**


1. Jansen, K.; Heirbaut, L.; Verkerk, R.; Cheng, J.D.; Joossens, J.; Cos, P.; Maes, L.; Lambeir, A.-M.; De Meester, I.; Augustyns, K.; et al. Extended Structure–Activity Relationship and Pharmacokinetic Investigation of (4-Quinolinoyl)Glycyl-2-Cyanopyrrolidine Inhibitors of Fibroblast Activation Protein (FAP). *J. Med. Chem.* **2014**, *57*, 3053–3074.
2. Lindner, T.; Loktev, A.; Altmann, A.; Giesel, F.; Kratochwil, C.; Debus, J.; Jäger, D.; Mier, W.; Haberkorn, U. Development of Quinoline-Based Theranostic Ligands for the Targeting of Fibroblast Activation Protein. *J. Nucl. Med.* **2018**, *59*, 1415–1422.
3. Thomas, A T; Gopalan, B, Lingam, V. S. P R, Lingam; Shah, DM. Preparation of glycinamide derivatives as dipeptidyl peptidase IV inhibitors. Patent, WO2005075426 A1 2005-08-18.
4. Lin, K.-S.; Pan, J.; Amouroux, G.; Turashvili, G.; Mesak, F.; Hundal-Jabal, N.; Pourghiasian, M.; Lau, J.; Jenni, S.; Aparicio, S.; et al. *In Vivo* Radioimaging of Bradykinin Receptor B1, a Widely Overexpressed Molecule in Human Cancer. *Cancer Res.* **2015**, *75*, 387–393.
